# Molecular Determinants of Substrate Selectivity of a Pneumococcal Rgg-Regulated Peptidase-Containing ABC Transporter

**DOI:** 10.1128/mBio.02502-19

**Published:** 2020-02-11

**Authors:** Charles Y. Wang, Jennifer S. Medlin, Don R. Nguyen, W. Miguel Disbennett, Suzanne Dawid

**Affiliations:** a Department of Microbiology and Immunology, University of Michigan, Ann Arbor, Michigan, USA; b Department of Pediatrics, University of Michigan, Ann Arbor, Michigan, USA; SJCRH; University of Mississippi Medical Center

**Keywords:** ABC transporters, *Streptococcus pneumoniae*, bacteriocins, quorum sensing

## Abstract

Peptidase-containing ABC transporters (PCATs) are a widely distributed family of transporters which secrete double-glycine (GG) peptides. In the opportunistic pathogen Streptococcus pneumoniae (pneumococcus), the PCATs ComAB and BlpAB have been shown to secrete quorum-sensing pheromones and bacteriocins related to the competence and pneumocin pathways. Here, we describe another pneumococcal PCAT, RtgAB, encoded by the *rtg* locus and found intact in 17% of strains. The Rgg/SHP-like quorum-sensing system RtgR/S, which uses a peptide pheromone with a distinctive Trp-X-Trp motif, regulates expression of the *rtg* locus and provides a competitive fitness advantage in a mouse model of nasopharyngeal colonization. RtgAB secretes a set of coregulated *rtg* GG peptides. ComAB and BlpAB, which share a substrate pool, do not secrete the *rtg* GG peptides. Similarly, RtgAB does not efficiently secrete ComAB/BlpAB substrates. We examined the molecular determinants of substrate selectivity between ComAB, BlpAB, and RtgAB and found that the GG peptide signal sequences contain all the information necessary to direct secretion through specific transporters. Secretion through ComAB and BlpAB depends largely on the identity of four conserved hydrophobic signal sequence residues previously implicated in substrate recognition by PCATs. In contrast, a motif situated at the N-terminal end of the signal sequence, found only in *rtg* GG peptides, directs secretion through RtgAB. These findings illustrate the complexity in predicting substrate-PCAT pairings by demonstrating specificity that is not dictated solely by signal sequence residues previously implicated in substrate recognition.

## Abstract (precis)

The export of peptides from the cell is a fundamental process carried out by all bacteria. One method of bacterial peptide export relies on a family of transporters called peptidase-containing ABC transporters (PCATs). PCATs export so-called GG peptides which carry out diverse functions, including cell-to-cell communication and interbacterial competition. In this work, we describe a PCAT-encoding genetic locus, *rtg*, in the pathogen Streptococcus pneumoniae (pneumococcus). The *rtg* locus is linked to increased competitive fitness advantage in a mouse model of nasopharyngeal colonization. We also describe how the *rtg* PCAT preferentially secretes a set of coregulated GG peptides but not GG peptides secreted by other pneumococcal PCATs. These findings illuminate a relatively understudied part of PCAT biology: how these transporters discriminate between different subsets of GG peptides. Ultimately, expanding our knowledge of PCATs will advance our understanding of the many microbial processes dependent on these transporters.

## INTRODUCTION

Export of polypeptides from their site of synthesis in the cytoplasm to the extracellular space is a fundamental physiological function of all cells. The secretome, the collection of all non-membrane-associated proteins secreted from the cell, may comprise up to 20% of an organism’s total proteome (1). Bacteria have evolved many different strategies for exporting proteins and peptides (2). One such strategy is the secretion of peptides using a family of ATP-binding cassette (ABC) transporters called peptidase-containing ABC transporters (PCATs).

PCATs are ABC transporters that contain characteristic N-terminal peptidase domains (PEPs). PEP belongs to the family of C39 cysteine proteases and is responsible for the proteolytic processing of substrates during transport (3). In Gram-positive bacteria, PCATs function either alone or with a single additional accessory protein (4). The most common function of PCATs is to assist in the biosynthesis of bacteriocins: antimicrobial peptides produced by bacteria to kill or otherwise inhibit the proliferation of other, usually closely related, bacteria (5). Some PCATs also promote cell-to-cell communication by secreting the peptide pheromones of Gram-positive quorum-sensing systems (6–10). In short, PCATs are widely distributed peptide transporters which play key roles in shaping how bacteria interact with each other.

Oftentimes, expression of PCATs is under the control of quorum-sensing systems. These regulatory systems rely on cell-to-cell signaling to induce and coordinate the expression of their target genes under specific conditions. One such mode of PCAT regulation is the Rgg/SHP pathway (11). Rgg is a family of transcription regulators found in many Gram-positive bacteria. In the genus *Streptococcus*, Rgg-family regulators are sometimes associated with short hydrophobic peptides (SHPs) (12). SHPs are small peptides which are exported by the PptAB transporter (13, 14) and processed into mature pheromones. The Ami oligopeptide importer then internalizes the pheromones back into the cell, where they bind to and modulate the activity of Rgg-family regulators (12). Besides PCATs and bacteriocin production, Rgg/SHP systems have been found to regulate diverse processes such as carbohydrate utilization (15), tissue invasion (13, 16), capsule production (16, 17), and biofilm formation (17, 18). A related group of Rgg regulators, the ComRs, are associated with SHP-like pheromones called ComS or XIP (SigX-inducing peptide) and control competence activation in some streptococcal species (19, 20).

In the Gram-positive opportunistic pathogen Streptococcus pneumoniae (pneumococcus), the PCATs ComAB and BlpAB secrete quorum-sensing pheromones that control two important cellular pathways: genetic competence (the ability to take up and incorporate extracellular DNA into the genome) and production of the major family of pneumococcal bacteriocins (Blp bacteriocins, or pneumocins) (6, 7, 21, 22). ComAB and BlpAB secrete the same GG peptides, including the competence- and pneumocin-inducing pheromones and the pneumocins (23–25). Substrate sharing between ComAB and BlpAB affects competence and pneumocin regulation and influences when and with what effectiveness naturally occurring BlpAB^+^ and BlpAB^−^ strains can employ pneumocin-mediated killing (25, 26).

The functional implications of the shared ComAB/BlpAB substrate pool highlight the need to better understand how different PCATs select their substrates. PCAT substrates contain N-terminal signal sequences (also called leader peptides) which terminate in a Gly-Gly (sometimes also Gly-Ala or Gly-Ser) motif (3). For this reason, they are referred to as double-glycine (GG) peptides. During transport, PEP cleaves the peptide bond following the GG motif to remove the signal sequence from the C-terminal mature peptide fragment (cargo peptide). The signal sequences of GG peptides bind to PEP of PCATs through hydrophobic interactions involving three or four conserved residues in the signal sequences (27–29). These residues are located at positions −4, −7, −12, and −15 relative to the scissile bond. The GG motif allows the substrate to fit in the narrow entrance to the active site of PEP and is also required for binding and cleavage (28, 30, 31). Besides these conserved residues, the signal sequences of different GG peptides are fairly divergent. Mutagenesis studies of several different PCAT-substrate pairs have largely failed to identify any contribution of these nonconserved residues to substrate-PEP binding (27–29).

While substantial progress has been made in uncovering the mechanisms that allow PCATs to recognize GG peptides, comparatively little is known about how or if PCATs discriminate between different GG peptides. In addition to ComAB and BlpAB from pneumococcus, multiple PCATs have been shown to process and/or secrete multiple peptides with distinct signal sequences, sometimes even those from different strains or species (28, 32–35). These data suggest that in general, PCATs are not particularly selective when it comes to choosing substrates.

In this work, we describe a previously uncharacterized locus in pneumococcus, *rtg*, which encodes the PCAT RtgAB and several GG peptides. This locus is regulated by the Rgg/SHP-like system RtgR/S, which provides a competitive fitness advantage during nasopharyngeal colonization. We demonstrate that RtgAB secretes the *rtg* GG peptides but not ComAB/BlpAB substrates and that ComAB or BlpAB cannot efficiently secrete the *rtg* GG peptides. Finally, we investigate the signal sequence determinants that selectively direct peptides toward either RtgAB or ComAB/BlpAB and show that a unique N-terminal motif is required for secretion by RtgAB. These findings shed light on how PCATs can use signal sequence motifs beyond the previously described conserved hydrophobic residues to distinguish different GG peptides.

## RESULTS

### Identification of an uncharacterized pneumococcal PCAT-encoding locus.

**FIG 1:**
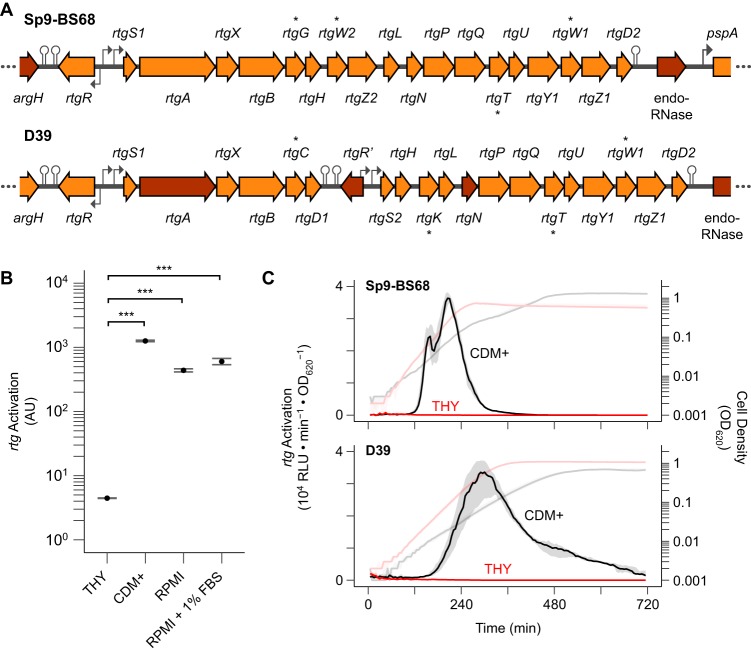
*rtg* is an actively regulated PCAT-encoding locus in pneumococcus. (A) Organization of *rtg* in strains Sp9-BS68 and D39. Block arrows represent genes. Dark shading indicates a pseudogene. Bent arrows represent promoters. Hairpins represent transcription terminators. GG peptide genes are marked with asterisks. (B) Activation of *rtg* in various growth media. A Sp9-BS68 P*_rtgA_*-*luc* reporter strain was grown in the indicated media, and luciferase activity was sampled when cells reached an OD_620_ of 0.2. The plotted values were normalized against cell density and the luciferase activity from a strain harboring constitutively expressed luciferase grown in the same medium to account for signal difference due to medium alone. Plots show means ± standard errors (SEs) from 3 independent experiments. ***, *P* < 0.001 by ANOVA with Tukey’s honestly significant difference (HSD); AU, arbitrary units. (C) Timing of *rtg* activation in THY and CDM+. Sp9-BS68 (top) and D39 (bottom) P*_rtgS1_*-*luc* reporter strains were grown in THY (red) and CDM+ (black) and monitored for *rtg* activation (dark, left *y* axis) and cell density (light, right *y* axis). Plots show medians (lines) and 25% to 75% quantiles (shading) from 12 wells pooled from 3 independent experiments.

As part of an effort to catalog the PCAT repertoire of S. pneumoniae, we searched pneumococcal genomes for putative PCAT genes that had not been previously described. One of the hits was *CGSSp9BS68_07257* (henceforth *07257*), a gene found in the clinical isolate Sp9-BS68 (36) (Fig. 1A). Upstream of *07257* is a gene oriented in the opposite direction predicted to encode an Rgg-family transcription regulator (12). We hypothesized that this regulator controls expression of *07257* and named the locus *rtg* (Rgg-regulated transporter of double-glycine peptides). We designated the transporter gene *rtgA* and the regulator gene *rtgR*. *rtgR* marks one end of the locus and is separated from a partially disrupted arginine biosynthesis cluster (37) by two transcription terminators. Downstream of *rtgA* are several genes arranged in a single operon. These include *rtgB*, which encodes a putative ComB/BlpB-like transport accessory protein, and the GG peptide genes *rtgG*, *rtgT*, *rtgW1*, and *rtgW2*. A transcription terminator separates the last gene, *rtgD2*, from a disrupted putative endoRNase gene and *pspA*. A different version of the *rtg* locus is found in the laboratory strain D39 (and its derivative, R6) but with a disrupted *rtgA* (Fig. 1A).

### The Rgg/SHP-like pheromone pair RtgR/RtgS regulates *rtg*.

We found that *rtg* expression is inhibited in mid-exponential phase during growth in the peptide-rich medium THY (Todd-Hewitt broth plus 0.5% yeast extract) but highly upregulated in the peptide-poor media CDM+ (38) and RPMI (Fig. 1B). The start of *rtg* activation in both Sp9-BS8 and D39 during growth in CDM+ occurs in early exponential phase at cell densities as low as an optical density at 620 nm (OD_620_) of 0.01 (Fig. 1C). In contrast, *rtg* stays inactive in THY throughout the exponential and stationary phases. We concluded from these data that *rtg* is actively regulated, most likely by RtgR. Since Rgg regulators are often associated with peptide pheromones, we searched for and found an open reading frame (ORF) in Sp9-BS68 between *rtgR* and *rtgA* predicted to encode a SHP-like pheromone. D39 has two copies of the candidate pheromone: one located between *rtgR* and *rtgA* and the other downstream of *rtgB*. We named the only copy of the ORF in Sp9-BS68 and the first copy in D39 (between *rtgR* and *rtgA*) *rtgS1* and the second copy in D39 *rtgS2* (Fig. 1A).

**FIG 2:**
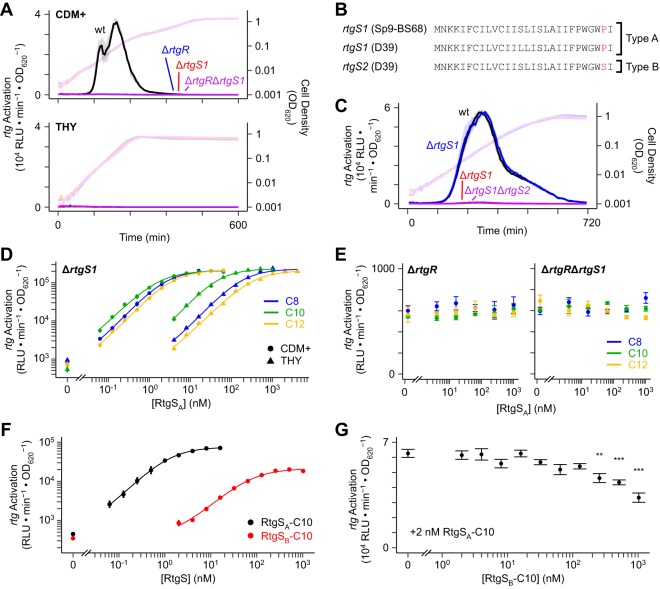
The Rgg/SHP-like RtgR/RtgS system regulates *rtg*. (A) *rtgR* and *rtgS1* are required for *rtg* activation in Sp9-BS68. Sp9-BS68 P*_rtgS1_*-*luc* reporters were grown in CDM+ or THY and monitored for *rtg* activation (dark, left *y* axis) and cell density (light, right *y* axis). Plots show medians (lines) and 25% to 75% quantiles (shading) from 12 wells pooled from 3 independent experiments. (B) Translated *rtgS* gene products from Sp9-BS68 and D39. The type-defining residues are highlighted in red. (C) *rtgS1* but not *rtgS2* is required for *rtg* activation in D39. D39 P*_rtgS1_*-*luc* reporters were grown in CDM+ and monitored for *rtg* activation (dark, left *y* axis) and cell density (light, right *y* axis). Plots show medians (lines) and 25% to 75% quantiles (shading) from 30 wells pooled from 3 independent experiments. C-terminal fragments of RtgS_A_ induce *rtg* in an RtgR-dependent manner. Sp9-BS68 P*_rtgS1_*-*luc* reporters were grown in CDM+ and THY (D) or CDM+ only (E) to an OD_620_ of 0.02 and treated with synthetic RtgS_A_ fragments. Response was defined as the maximum observed P*_rtgS1_* activity within 60 min of treatment. Data in panel D were fitted to the four-parameter Hill model (curves). Plotted data points represent means ± SEs from 3 independent experiments. (F) RtgS_B_ is a partial agonist at the *rtg* locus. A D39 Δ*rtgS1* Δ*rtgS2* P*_rtgS1_*-*luc* reporter was grown in CDM+ to an OD_620_ of 0.02 and treated with synthetic RtgS fragments. Response was defined as the maximum observed P*_rtgS1_* activity within 60 min of treatment. Data were fitted to the four-parameter Hill model (curves). Plotted data points represent means ± SEs from 3 independent experiments. (G) High concentrations of RtgS_B_-C10 antagonize RtgS_A_-C10. A D39 Δ*rtgS1* Δ*rtgS2* P*_rtgS1_*-*luc* reporter was grown in CDM+ to an OD_620_ of 0.02 and treated simultaneously with 2 nM RtgS_A_-C10 and various concentrations of RtgS_B_-C10. Response was defined as the maximum observed P*_rtgS1_* activity within 120 min of treatment. Plotted data points represent means ± SEs from 3 independent experiments. **, *P* < 0.01; ***, *P* < 0.001 versus 0 nM RtgS_B_-C10 by ANOVA with Dunnett’s correction for multiple comparisons.

Having identified a putative Rgg/SHP-like regulatory system, we sought to define the contributions of RtgR and RtgS to *rtg* regulation through deletional analysis. We monitored *rtg* activation in Sp9-BS68 Δ*rtgS1*, Δ*rtgR*, and Δ*rtgS1* Δ*rtgR* strains during growth in CDM+ and THY (Fig. 2A). None of the mutants showed signs of *rtg* activation in either medium, indicating that both RtgR and RtgS promote and are required for *rtg* activation. In D39, *rtgS1* encodes a peptide with a single amino acid change (S14L) compared to RtgS from Sp9-BS68, and *rtgS2* encodes a peptide with a different single amino acid change (P27S) (Fig. 2B). We found that the D39 Δ*rtgS1* and Δ*rtgS1* Δ*rtgS2* mutants failed to activate *rtg* in CDM+, while the Δ*rtgS2* mutant was indistinguishable from the wild-type strain (Fig. 2C). This suggested that the P27S substitution in the *rtgS2* product prevents it from activating *rtg*, while the S14L substitution in the *rtgS1* product does not appreciably affect signaling. Therefore, we classified the *rtgS1* product in both Sp9-BS68 and D39 as type A pheromone (RtgS_A_) and the *rtgS2* product in D39 as type B (RtgS_B_).

To confirm that RtgS is the specific pheromone inducer of *rtg*, we performed dose-response assays using synthetic peptides corresponding to the C-terminal 8, 10, and 12 residues of RtgS_A_ (RtgS_A_-C8, RtgS_A_-C10, and RtgS_A_-C12, respectively). All three synthetic peptides induced expression from the Sp9-BS68 *rtgS1* promoter in both CDM+ and THY, though the curves for the latter were shifted to the right in a manner consistent with pure competitive inhibition (Fig. 2D; Table 1). We also confirmed that *rtg* induction by synthetic RtgS requires RtgR (Fig. 2E). In D39, RtgS_A_-C10 induces *rtg* similarly to Sp9-BS68, whereas RtgS_B_-C10 acts as a partial agonist with a 55-fold larger 50% effective concentration (EC_50_) value than RtgS_A_-C10 (Fig. 2F; Table 1). Therefore, the Pro-to-Ser substitution in RtgS_B_ interferes with signaling at a step following pheromone secretion. Although partial agonists can act as competitive antagonists of full agonists, we did not observe an inhibitory phenotype associated with RtgS_B_ during natural *rtg* activation (Fig. 2C), and competitive dose-response assays showed that RtgS_B_-C10 only antagonizes RtgS_A_-C10 at likely supraphysiological concentrations (≥256 nM) (Fig. 2G).

**TABLE 1:** RtgS dose-response parameters

Strain	Growth medium	Peptide	EC_50_ (nM)^*a*^	Maximal response (10^3^ RLU·min^−1^·OD_620_^−1^)^*a*^
Sp9-BS68^*b*^	CDM+	RtgS_A_-C8	2.53 ± 0.25	212.2 ± 9.4
		RtgS_A_-C10	1.63 ± 0.19	205.7 ± 9.1
		RtgS_A_-C12	3.51 ± 0.28	213.9 ± 8.0
	THY	RtgS_A_-C8	184 ± 15	229.7 ± 8.6
		RtgS_A_-C10	47.4 ± 6.9	232.4 ± 14.3
		RtgS_A_-C12	315 ± 42	210.1 ± 13.1
D39^*c*^	CDM+	RtgS_A_-C10	1.25 ± 0.30	75.1 ± 8.1
		RtgS_B_-C10	68.8 ± 11.5	21.2 ± 1.5

a Means ± SEs; derived from fitting data to Hill model.

b Δ*rtgS1*.

c Δ*rtgS1* Δ*rtgS2*.

Next, we determined that, consistent with previously described Rgg/SHP systems, *rtg* activation requires both the Ami importer and the PptAB transporter (see Fig. S1A in the supplemental material). We also showed that response to exogenous RtgS treatment requires Ami but not PptAB (Fig. S1B), consistent with their respective roles as pheromone importer and exporter.

10.1128/mBio.02502-19.1 FIG S1 Ami and PptAB are required for RtgR/S signaling. (A) Both Ami and PptAB are required for natural *rtg* autoinduction. Sp9-BS68 P*_rtgS1_*-*luc* reporters were grown in CDM+ and monitored for *rtg* activation (dark, left *y* axis) and cell density (light, right *y* axis). Plots show medians (lines) and 25% to 75% quantiles (shading) from 30 wells per strain pooled from 3 independent experiments. (B) Exogenous RtgS treatment rescues *rtg* activation defect in the Δ*pptAB* mutant but not the Δ*amiCD* mutant. Sp9-BS68 P*_rtgS1_*-*luc* reporters were grown in CDM+ to an OD_620_ of 0.02 and treated with RtgS_A_-C10. Response was defined as the maximum observed P*_rtgS1_* activity within 60 min of treatment. Plotted data points represent means ± SEs from 3 independent experiments. Download FIG S1, PDF file, 0.6 MB. Copyright © 2020 Wang et al. 2020 Wang et al. This content is distributed under the terms of the Creative Commons Attribution 4.0 International license.

Finally, mutagenesis of the Sp9-BS68 *rtgS1* promoter region revealed the presence of two nearly identical promoters, each contributing partially to *rtgS1* expression (see Fig. S2A and B). We also identified an inverted repeat found in both promoters which is required for RtgS-induced expression and likely represents the RtgR binding site (Fig. S2A and C).

10.1128/mBio.02502-19.2 FIG S2 Two promoters, P1 and P2, both with putative RtgR binding sites, contribute to *rtgS1* expression. (A) Promoter region of *rtgR* and *rtgS1* in Sp9-BS68. Inverted repeats (IR) are underlined; −10 elements are marked with dotted arrows. Start sites of *rtgR* and *rtgS1* are marked with solid arrows. Bent arrow indicates span of P2 promoter construct used in panels B and C. Colored numbered boxes indicate mutations used in panel C; black letters represent the original nucleotides while colored letters represent the mutated nucleotides. (B) P1 and P2 each partially contribute to *rtgS1* expression. Sp9-BS68 Δ*rtgS1* P*_rtgS1_*-*luc* reporters were grown in CDM+ to an OD_620_ of 0.02 and treated with synthetic RtgS_A_-C10. Response was defined as the maximum observed P*_rtgS1_* activity within 60 min of treatment. Plotted data points represent means ± SEs from 3 independent experiments. *, *P* < 0.05; **, *P* < 0.01; ***, *P* < 0.001 between P1+P2 and P2 reporters at each dose by ANOVA followed by Holm-corrected posttests. (C) IR1 is required for RtgS-induced *rtg* activation. Sp9-BS68 Δ*rtgS1* P*_rtgS1_*-*luc* reporters were grown in CDM+ to an OD_620_ of 0.02 and treated with synthetic RtgS_A_-C10. Except for the Δ*rtgR* strain, *luc* in each reporter was fused to P2 only. Response was defined as the maximum observed P*_rtgS1_* activity within 60 min of treatment. Plotted data points represent means ± SEs from 3 independent experiments. Download FIG S2, PDF file, 0.5 MB. Copyright © 2020 Wang et al. 2020 Wang et al. This content is distributed under the terms of the Creative Commons Attribution 4.0 International license.

### RtgAB secretes *rtg*-encoded GG peptides.

**FIG 3:**
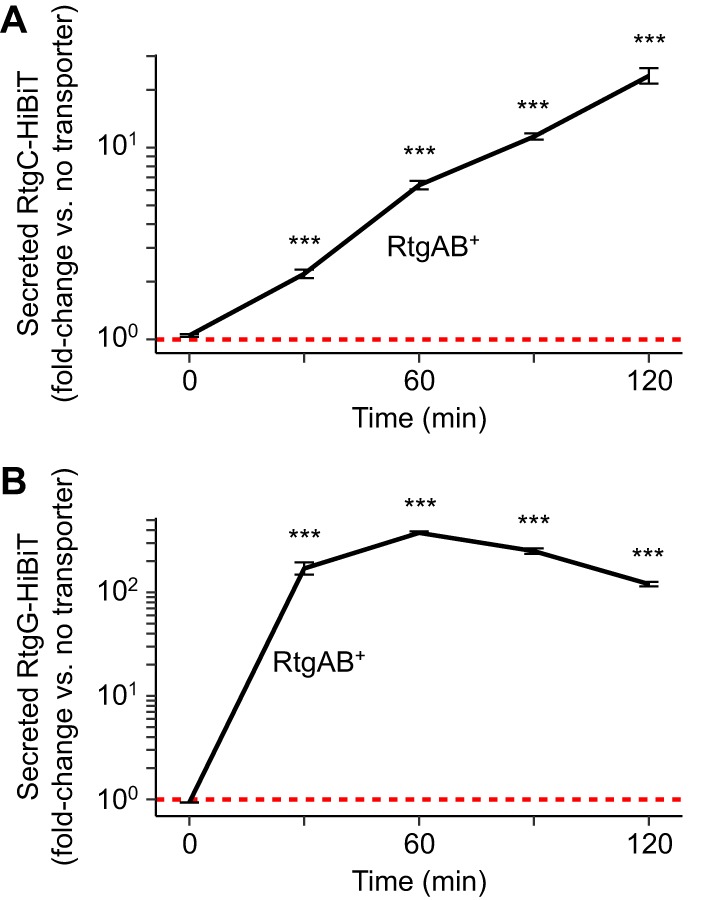
RtgAB secretes RtgC and RtgG. R6 ComAB^−^/BlpAB^−^/RtgAB^−^ and ComAB^−^/BlpAB^−^/RtgAB^+^ strains expressing RtgC-HiBiT (A) or RtgG-HiBiT (B) were grown in CDM+ to an OD_620_ of 0.05 and treated with 200 ng/ml CSP, 200 ng/ml BlpC, and 20 nM RtgS_A_-C10. CSP and BlpC were added to allow for comparison with ComAB^+^ and BlpAB^+^ strains in later experiments. Samples were taken every 30 min, and extracellular HiBiT signal was quantified. Data are presented as fold change values relative to the ComAB^−^/BlpAB^−^/RtgAB^−^ control. Red dashed lines represent fold change = 1 (no difference versus the control). Plots show means ± SEs from 3 independent experiments. ***, *P* < 0.001 versus ComAB^−^/BlpAB^−^/RtgAB^−^ by ANOVA with Tukey’s HSD.

Sp9-BS68 and D39 both harbor four putative GG peptides at the *rtg* locus (Fig. 1A). To determine whether RtgAB secretes the *rtg* GG peptides, we employed a HiBiT tag-based peptide secretion assay (25). We constructed autoinducing-deficient Δ*rtgS1* Δ*rtgS2* R6 strains which harbor HiBiT-tagged *rtgC* (from D39) or *rtgG* (from Sp9-BS68) placed downstream of *rtgB* in RtgAB^−^ (native R6 *rtgA* pseudogene) and RtgAB^+^ (pseudogene-repaired) backgrounds. These strains were also ComAB^−^ and BlpAB^−^ in order to remove the possibility of peptide secretion through these other PCATs. Upon RtgS-C10 treatment in CDM+, levels of extracellular RtgC-HiBiT and RtgG-HiBiT in the RtgAB^+^ cultures increased 26- and 376-fold, respectively, compared to their levels in the RtgAB^−^ cultures (Fig. 3). From these data, we conclude that RtgAB secretes the *rtg* GG peptides.

### The *rtg* locus exhibits extensive variation across different pneumococcal strains.

To catalog the diversity found at *rtg*, we conducted a survey of the locus in a collection of pneumococcal clinical isolates from Massachusetts, USA (39). After removing genomes in which *rtg* spans multiple contigs or when conserved genes flanking *rtg* could not be found, we were left with 318 of 616 strains, all of which encoded at least one *rtg* gene. We analyzed the *rtg* loci from these 318 strains and clustered them into 23 groups based on overall architecture (see Fig. S3A). Across all 318 loci, we found 24 unique *rtg* genes (Table 2), including eight putative GG peptides which share highly conserved signal sequences (Fig. S3B). Searching for these genes in the full collection revealed that 615 of 616 strains had some version of the *rtg* locus. Next, we analyzed the variation in RtgS among the 318 filtered strains. We found at least one copy of *rtgS* in each strain. Because duplication of *rtgS* is common, we assigned the name *rtgS1* to any copy located next to *rtgR* and the name *rtgS2* to any copy located next to *rtgR′*. Based on the identity of the penultimate residue in the translated peptide, which we have shown is important for signaling activity, we catalogued a total of three pheromone types: types A (Pro), B (Ser), and C (Leu). Only two other positions in the last 12 residues of RtgS showed variation: Ala/Val at position −10 from the C terminus and Ile/Val at position −8. The functional significance of these other polymorphisms is unknown. Finally, we analyzed each strain’s RtgAB status and found 5% of strains carry unambiguously intact *rtgA* and *rtgB*. Another 12% carry intact *rtgB* and a version of *rtgA* with a start codon mutation (ATG>ATT) but that is otherwise intact. We determined that a strain with the ATG>ATT mutation still produces functional RtgAB, likely by using an alternative start site, and suffers only a minor reduction in secretion capacity compared to that of a strain with fully intact *rtgA* (see Fig. S4). Therefore, 17% of strains encode a functional RtgAB.

**TABLE 2:** Genes of the *rtg* locus

Gene	Product	Frequency (%)^*a*^
*rtgA*		
Intact	Peptidase-containing ABC transporter	5.0
Start codon mutation only	Peptidase-containing ABC transporter	14.5
Disrupted	Peptidase-containing ABC transporter, truncated	59.1
*rtgB*		
Intact	Putative transport accessory protein	18.6
Disrupted	Putative transport accessory protein, truncated	2.5
*rtgC*	GG peptide	15.1
*rtgD*	Hypothetical protein	99.7
*rtgE*	GG peptide	7.2
*rtgG*	GG peptide	4.1
*rtgH*	Hypothetical protein	40.6
*rtgK*	GG peptide	41.2
*rtgL*	Hypothetical protein	83.3
*rtgM*	GG peptide	59.7
*rtgN*	Hypothetical protein	82.4
*rtgP*	Putative integral membrane protein	83.3
*rtgQ*	Putative integral membrane protein	83.3
*rtgR*	Rgg-family transcription regulator	78.6
*rtgR′*	Rgg-family transcription regulator, truncated	36.5
*rtgS*		
Type A	SHP-like pheromone, type A	42.5
Type B	SHP-like pheromone, type B	67.6
Type C	SHP-like pheromone, type C	0.6
*rtgT*	GG peptide	83.0
*rtgU*	Hypothetical protein	83.0
*rtgV*	GG peptide; probable fusion of RtgW and RtgZ	35.5
*rtgW*	GG peptide	83.0
*rtgX*	GyrI-like hypothetical protein	21.1
*rtgY*	Putative integral membrane protein	88.7
*rtgZ*	Putative integral membrane protein	99.7

a In 318 genomes from the Massachusetts collection with fully sequenced gapless *rtg* loci. May include pseudogenes unless stated otherwise.

10.1128/mBio.02502-19.3 FIG S3 Diversity of fully sequenced *rtg* loci and GG peptide signal sequences in the Massachusetts pneumococcal isolate collection. (A) *rtg* loci from a filtered set of 318 strains from the Massachusetts isolate collection with gapless sequence coverage of the *rtg* genomic locale were clustered into 23 groups based on gene content and synteny. The organization of *rtg* in each of these groups is depicted here. Block arrows represent genes and are color coded according to their predicted function/classification. Genes not explicitly marked as pseudogenes may still be present as pseudogenes in a subset of strains within a group. The number and percentage of strains (out of 318) belonging to each group is displayed on the right. Regarding the classification of the strains used in this work: D39/R6 is a member of group 20a (but with a disrupted *rtgA* gene) and Sp9-BS68 is a member of group 1. (B) Alignment of representative signal sequences found in predicted *rtg* GG peptides. Asterisks mark peptides that were tested in this work. Amino acids in red were specifically assessed in this work. Groups refer to the classification in panel A. Download FIG S3, PDF file, 0.8 MB. Copyright © 2020 Wang et al. 2020 Wang et al. This content is distributed under the terms of the Creative Commons Attribution 4.0 International license.

10.1128/mBio.02502-19.4 FIG S4 Strains with the *rtgA_ATG>ATT_* mutation still produce functional RtgAB. R6 ComAB^−^/BlpAB^−^/RtgAB^−^ and RtgAB^+^ strains with (blue) or without (black) the ATG>ATT mutation in *rtgA* expressing RtgG-HiBiT were grown in CDM+ to an OD_620_ of 0.05 and treated with 200 ng/ml CSP, 200 ng/ml BlpC, and 20 nM RtgS_A_-C10. Samples were taken every 30 min, and extracellular HiBiT signal was quantified (left). Data are presented as fold change values relative to the ComAB^−^/BlpAB^−^/RtgAB^−^ control. Red dashed line represents fold change = 1 (no difference versus the control). At the 120-min timepoint, intracellular peptide content was also quantified (right). Plot shows means ± SEs from 3 independent experiments. Download FIG S4, PDF file, 0.4 MB. Copyright © 2020 Wang et al. 2020 Wang et al. This content is distributed under the terms of the Creative Commons Attribution 4.0 International license.

### Active RtgR/S confers a competitive fitness advantage during nasopharyngeal colonization.

**FIG 4:**
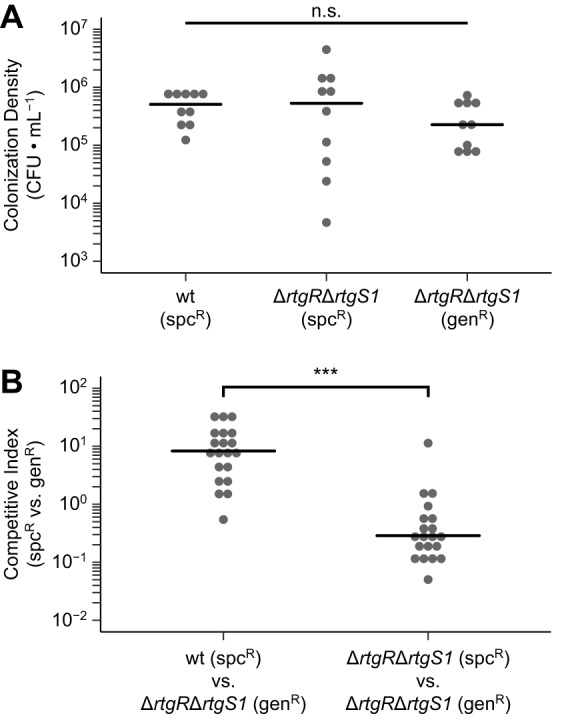
Active RtgR/S provides a competitive fitness advantage during nasopharyngeal colonization. (A) Sp9-BS68 strains were singly inoculated into the nasopharynx of 5- to 7-week-old female BALB/c mice. At 3 days postinoculation, colonization density was sampled by nasal wash. Black bars represent medians. *N* = 10 mice per strain pooled from 2 independent experiments. n.s., not significant by Kruskal-Wallis test. (B) Pairs of Sp9-BS68 strains were coinoculated into the nasopharynxes of 5- to 7-week-old female BALB/c mice. At 3 days postinoculation, colonization density was sampled by nasal wash, and competitive indices were calculated. Black bars represent medians. *N* = 20 mice per competition pooled from 2 independent experiments. ***, *P* < 0.001 by Mann-Whitney test.

To determine the biological role of *rtg*, we tested the effect of a regulatory deletion on colonization of the nasopharynx, the natural niche of pneumococcus. Despite similar levels of colonization between the wild-type and Δ*rtgR* Δ*rtgS1* strains in singly inoculated mice at 3 days postinoculation (Fig. 4A), the wild-type strain outcompeted the mutant in coinoculated mice (Fig. 4B). These data suggest that RtgR/S is active during nasopharyngeal colonization and show that active RtgR/S provides a fitness advantage over RtgR/S-inactive strains during cocolonization.

### RtgAB and ComAB/BlpAB preferentially secrete different sets of peptides.

**FIG 5:**
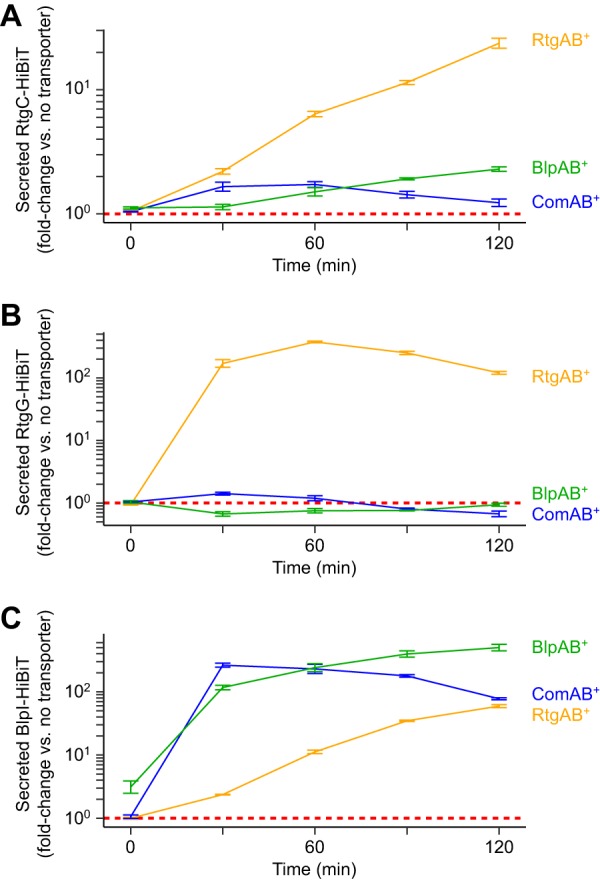
RtgAB secretes different GG peptides from ComAB and BlpAB. R6 ComAB^−^/BlpAB^−^/RtgAB^−^ and single-transporter-positive ComAB^+^, BlpAB^+^, and RtgAB^+^ strains expressing RtgC-HiBiT (A), RtgG-HiBiT (B), or BlpI-HiBiT (C) were grown in CDM+ to an OD_620_ of 0.05 and treated with 200 ng/ml CSP, 200 ng/ml BlpC, and 20 nM RtgS_A_-C10. Samples were taken every 30 min, and extracellular HiBiT signal was quantified. Data are presented as fold change values relative to the ComAB^−^/BlpAB^−^/RtgAB^−^ control. Red dashed lines represent fold change = 1 (no difference versus the control). Plots show means ± SEs from 3 independent experiments.

The pneumococcal PCATs ComAB and BlpAB secrete the same diverse set of GG peptides (23–25). Therefore, we wondered if ComAB and BlpAB could also secrete the *rtg* GG peptides and if RtgAB is similarly promiscuous and could secrete ComAB/BlpAB substrates. We repeated the RtgC-HiBiT and RtgG-HiBiT secretion assays with ComAB^+^ and BlpAB^+^ strains, using treatment with the *com* and *blp* pheromones competence-stimulating peptide (CSP) and BlpC, respectively, to induce their expression. ComAB and BlpAB secrete markedly reduced amounts of RtgC-HiBiT and RtgG-HiBiT compared to the amounts secreted by RtgAB (Fig. 5A and B). To determine if RtgAB could secrete a ComAB/BlpAB substrate, we assayed secretion of a HiBiT-tagged version of the BlpI bacteriocin driven by its native promoter. RtgAB secretes roughly 10-fold less BlpI-HiBiT than BlpAB (Fig. 5C). Therefore, RtgAB and ComAB/BlpAB do not efficiently cross-secrete each other’s substrates. Consistent with this, RtgAB^+^ strains do not show differences in *com* or *blp* activation compared to that of RtgAB^−^ strains during growth in CDM+ (see Fig. S5). Under these conditions, *rtg* is expected to turn on before *com* or *blp* in every strain background. Thus, even when RtgAB is highly expressed, it secretes too little CSP and BlpC to affect the timing of *com* and *blp* activation.

10.1128/mBio.02502-19.5 FIG S5 RtgAB does not affect the timing of *com* or *blp* activation during growth in CDM+. D39 P*_comA_*-*Nluc* P_BIR_-*RFluc* dual reporters were grown in CDM+ and monitored for *com* (top) and *blp* (bottom) activation. Data were fit to the Kaplan-Meier estimator. Wells that did not experience an activation event before cells reached their maximum density were censored (crosses). *N* = 32 (ComAB^+^/BlpAB^+^ strains) or 16 (all other strains) wells per strain pooled from 4 independent experiments. All comparisons between RtgAB^+^ and RtgAB^−^ strains with identical ComAB/BlpAB phenotypes (except in the ComAB^−^/BlpAB^−^ background, for which all data points were censored) were not significant (*P* ≥ 0.05) by log-rank test with Holm correction. Download FIG S5, PDF file, 0.5 MB. Copyright © 2020 Wang et al. 2020 Wang et al. This content is distributed under the terms of the Creative Commons Attribution 4.0 International license.

### RtgAB and ComAB/BlpAB recognize their substrates through different signal sequence motifs.

**FIG 6:**
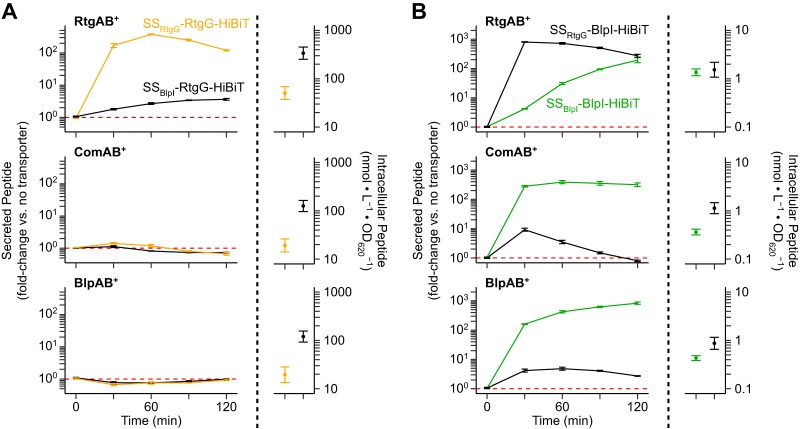
RtgAB, ComAB, and BlpAB recognize GG peptides through their N-terminal signal sequences. R6 ComAB^−^/BlpAB^−^/RtgAB^−^ and single-transporter-positive ComAB^+^, BlpAB^+^, and RtgAB^+^ strains expressing signal sequence-swapped RtgG-HiBiT cargo peptide (A) or BlpI-HiBiT cargo peptide (B) were grown in CDM+ to an OD_620_ of 0.05 and treated with 200 ng/ml CSP, 200 ng/ml BlpC, and 20 nM RtgS_A_-C10. Samples were taken every 30 min, and extracellular HiBiT signal was quantified (left). Data are presented as fold change values relative to the ComAB^−^/BlpAB^−^/RtgAB^−^ control. Red dashed lines represent fold change = 1 (no difference versus the control). At the 120-min time point, intracellular peptide content was also quantified (right). Plots show means ± SEs from 3 independent experiments.

Given that we had found RtgAB and ComAB/BlpAB do not share the same substrate pool, we explored how the transporters discriminate between substrate and nonsubstrate GG peptides. We showed that the BlpI signal sequence (SS_BlpI_) prevents secretion of the RtgG cargo peptide through RtgAB (Fig. 6A). However, it did not promote secretion of RtgG through ComAB/BlpAB, suggesting an incompatibility between the cargo peptide and these two transporters. On the other hand, the RtgG signal sequence (SS_RtgG_) promotes secretion of the BlpI cargo peptide through RtgAB while preventing its secretion through ComAB and BlpAB (Fig. 6B).

To rule out the possibility of differences in peptide expression being solely responsible for the secretion differences, we also measured the amount of intracellular peptide in each assay (Fig. 6A and B; right-hand graphs). The signal sequence swaps affected intracellular peptide levels. However, these intracellular differences cannot account for the observed changes in secretion; higher intracellular levels did not correlate with more secretion, and while intracellular levels of the same peptide were relatively consistent across different strains (RtgAB^+^ versus ComAB^+^ versus BlpAB^+^), secretion was not. Thus, the observed changes in secretion between the different peptides most likely reflect differences in peptide-transporter interactions.

In conclusion, while cargo peptide can dictate transporter compatibility in some cases, the signal sequences of GG peptides still contain all the necessary information to direct secretion of their own cargo peptides through the proper transporters. For all future assays, we used BlpI as the cargo peptide, since it can be secreted by all three transporters given the correct signal sequence.

**FIG 7:**
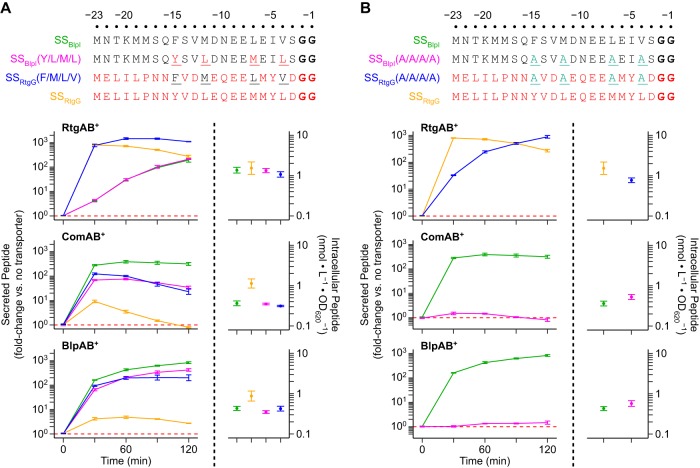
Specific GG peptide signal sequence residues at positions −15, −12, −7, and −4 modulate secretion by ComAB and BlpAB but not RtgAB. R6 ComAB^−^/BlpAB^−^/RtgAB^−^ and single-transporter-positive ComAB^+^, BlpAB^+^, and RtgAB^+^ strains expressing BlpI or RtgG signal sequences with residue swaps (A) or alanine substitutions (B) at positions −15, −12, −7, and −4 (top, mutated positions are underlined) fused to BlpI-HiBiT cargo peptide were grown in CDM+ to an OD_620_ of 0.05 and treated with 200 ng/ml CSP, 200 ng/ml BlpC, and 20 nM RtgS_A_-C10. Samples were taken every 30 min, and extracellular HiBiT signal was quantified (left). Data are presented as fold change values relative to the ComAB^−^/BlpAB^−^/RtgAB^−^ control. Red dashed lines represent fold change = 1 (no difference versus the control). At the 120-min time point, intracellular peptide content was also quantified (right). Plots show means ± SEs from 3 independent experiments.

Next, we searched for the specific signal sequence residues involved in transport selectivity. We found that secretion of peptide through ComAB/BlpAB depends on the identities of the residues at the conserved signal sequence positions −15, −12, −7, and −4. These positions were previously implicated in substrate recognition by PCATs (27, 28, 30). The combination of the four residues at these positions from SS_BlpI_ (F/M/L/V) introduced into SS_RtgG_ promote secretion through ComAB/BlpAB, although they were not strictly required for secretion in the context of SS_BlpI_ (Fig. 7A). The complementary association did not hold for RtgAB-mediated secretion, in that the four residues from SS_RtgG_ (Y/L/M/L) were neither necessary nor sufficient for secretion through RtgAB (Fig. 7A). Additionally, alanine substitutions at all four positions in SS_RtgG_ only partially impeded secretion through RtgAB, but the same substitutions in SS_BlpI_ prevented secretion through ComAB and BlpAB almost entirely (Fig. 7B).

### A specific motif at the N-terminal ends of *rtg* GG peptide signal sequences promotes secretion through RtgAB.

**FIG 8:**
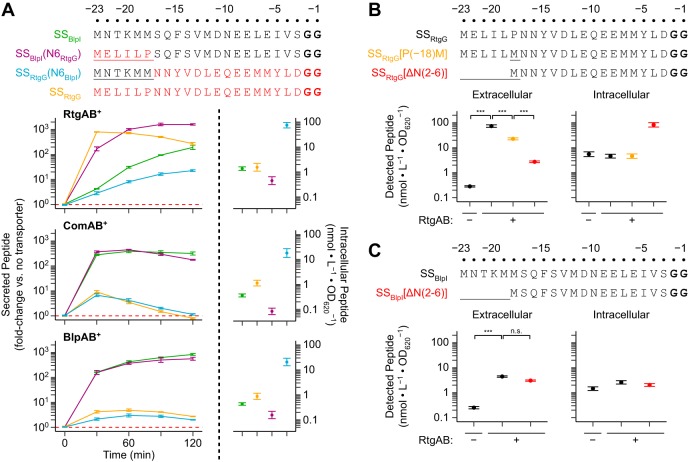
A unique motif found at the N-terminal ends of the *rtg* GG peptides promotes secretion by RtgAB. (A) R6 ComAB^−^/BlpAB^−^/RtgAB^−^ and single-transporter-positive ComAB^+^, BlpAB^+^, and RtgAB^+^ strains expressing BlpI or RtgG signal sequences with residue swaps at positions −22 to −18 (top, mutated positions are underlined) fused to BlpI-HiBiT cargo peptide were grown in CDM+ to an OD_620_ of 0.05 and treated with 200 ng/ml CSP, 200 ng/ml BlpC, and 20 nM RtgS_A_-C10. Samples were taken every 30 min, and extracellular HiBiT signal was quantified (left). Data are presented as fold change values relative to the ComAB^−^/BlpAB^−^/RtgAB^−^ control. Red dashed lines represent fold change = 1 (no difference versus the control). At the 120-min time point, intracellular peptide content was also quantified (right). Plots show means ± SEs from 3 independent experiments. R6 ComAB^−^/BlpAB^−^/RtgAB^−^ and RtgAB^+^ strains expressing mutated SS_RtgG_ (B) or SS_BlpI_ (C) (top, mutated positions are underlined) fused to BlpI-HiBiT cargo peptide were grown and treated with pheromone as in panel A. Extracellular (left) and intracellular (right) HiBiT signals were quantified at 60 min posttreatment. Plots show means ± SEs from 3 independent experiments. n.s., not significant; ***, *P* < 0.001 by ANOVA with Tukey’s HSD.

To identify the signal sequence residues that promote secretion through RtgAB, we turned our attention to the N-terminal ends of the signal sequences, which are conserved in *rtg* GG peptides but not in ComAB/BlpAB substrates. Residue swaps at positions −22 to −18 in SS_RtgG_ and SS_BlpI_ demonstrated that secretion through RtgAB, but not ComAB or BlpAB, depends on specific signal sequence residues in this region (Fig. 8A). The P(−18)M substitution in SS_RtgG_ modestly decreased secretion through RtgAB, and removal of all residues on the N-terminal side of this substitution further decreased secretion (Fig. 8B). Meanwhile, removal of the residues at the same positions from SS_BlpI_ did not change secretion through RtgAB (Fig. 8C). These data indicate that the residues in this region in SS_RtgG_ were selected to interact with RtgAB rather than to avoid steric clash. Alanine scanning mutagenesis of the −22 to −19 region of SS_RtgG_ revealed that secretion through RtgAB was not sensitive to mutation at any single site (see Fig. S6). These data can be explained by multiple redundant residues mediating the interactions in this region or the interactions being tolerant to alanine substitution. We conclude that RtgAB recognizes *rtg* GG peptides through interactions involving the signal sequence residues in the −22 to −18 region. At the same time, RtgAB’s substrate recognition mechanism has evolved to be less reliant than that of ComAB or BlpAB on interactions with the hydrophobic signal sequence residues at positions −15, −12, −7, and −4.

10.1128/mBio.02502-19.6 FIG S6 Single alanine substitutions in the N-terminal region of SS_RtgG_ do not decrease secretion through RtgAB. R6 ComAB^−^/BlpAB^−^/RtgAB^−^ and RtgAB^+^ strains expressing mutated SS_RtgG_ fused to BlpI-HiBiT cargo peptide were grown in CDM+ to an OD_620_ of 0.05 and treated with 200 ng/ml CSP, 200 ng/ml BlpC, and 20 nM RtgS_A_-C10. Extracellular and intracellular HiBiT signals were quantified at 60 min posttreatment. Corrected secreted peptide levels were calculated by normalizing extracellular signals to intracellular signals. Data are presented as fold change values relative to the ComAB^−^/BlpAB^−^/RtgAB^−^ control strain with wild-type SS_RtgG_. Plots show means ± SEs from 3 independent experiments. n.s., not significant; ***, *P* < 0.001 by ANOVA with Dunnett’s posttest. Download FIG S6, PDF file, 0.4 MB. Copyright © 2020 Wang et al. 2020 Wang et al. This content is distributed under the terms of the Creative Commons Attribution 4.0 International license.

## DISCUSSION

In this work, we have characterized the PCAT-encoding locus *rtg* and shown it is regulated by the RtgR/S system. RtgR/RtgS belongs to a family of regulatory systems found in streptococci that includes the Rgg/SHP and ComR/S systems (12). Rgg/SHP and ComR/S circuits can act as either cell density-dependent quorum-sensing systems (12) or timing devices (40). Our data suggest RtgR/S behaves like the former (see Fig. S7 in the supplemental material). A purely intracellular signaling pathway has been reported for XIP in Streptococcus mutans (14, 41). Such a pathway is unlikely to exist for RtgR/S, since *rtg* autoinduction requires both PptAB and Ami (Fig. S1A). While the RtgS pheromone is similar to the previously described SHP and ComS/XIP pheromones, it also differs from these other pheromone classes in important ways. RtgS lacks the conserved aspartate or glutamate residue characteristic of SHPs and is divergently transcribed from its regulator unlike ComS (12). However, RtgS does contain a Trp-Gly-Trp motif near the C terminus which bears resemblance to the Trp-Trp motif found in some XIPs (12, 20). RtgR is phylogenetically closer to the ComRs than SHP-associated Rgg regulators but does not cluster with either group (12). Using a published list of Rgg regulators (12), we found two RtgR-like regulators associated with Trp-X-Trp (WxW) motif-containing pheromones: SPD_1518 (Rgg1518) from S. pneumoniae D39 and SSA_2251 from Streptococcus sanguinis SK36 (predicted unprocessed pheromone sequences, MGFKKYLKNLPKNSGFLIWSWIQLIWFETWFWG and MKKIVYNLILLAVTSIVTTSVFPWWWLWW, respectively). Expression analysis of the pheromone operon associated with *rgg1518* using PneumoExpress (42) revealed that the pheromone and genes *SPD_1513* to *SPD_1517* are specifically upregulated under the same conditions that result in upregulation of *rtg*. Therefore, the Rgg1518 system is likely functional. We propose that RtgR/S and other Rgg/WxW pheromone pairs constitute a distinct group of Rgg regulatory systems. We leave the work of characterizing the members of this group and the pathways they regulate to future studies.

10.1128/mBio.02502-19.7 FIG S7 Timing of *rtg* activation depends on cell density. The wild-type Sp9-BS68 P*_rtgS1_*-*luc* reporter was inoculated into CDM+ at different starting densities, grown, and monitored for *rtg* activation (dark, left *y* axis) and cell density (light, right *y* axis). Plot shows median (line) and 25% to 75% quantiles (shading). *N *= 30 wells pooled from 3 independent experiments for each of the starting densities of 0.003 and 0.0015. *N* = 29 wells pooled from 3 independent experiments for starting density of 0.00075; data from one well was discarded due to lack of growth. Download FIG S7, PDF file, 0.5 MB. Copyright © 2020 Wang et al. 2020 Wang et al. This content is distributed under the terms of the Creative Commons Attribution 4.0 International license.

We showed that in the RtgAB^+^ strain Sp9-BS68, the ability to activate the RtgR/S system confers a fitness advantage during competitive colonization of the nasopharynx. While 78% of strains are predicted to harbor a functional RtgR and therefore can respond to pheromone, only 17% of strains are RtgAB^+^. Most RtgAB^−^ strains still harbor at least one *rtg* GG peptide but have no obvious means with which to secrete them, since they are not secreted by the other two PCATs commonly found in pneumococcus, ComAB and BlpAB. We have been unable to determine the function of the *rtg* GG peptides, but we speculate that they are bacteriocins. The reasons for this are that bacteriocin secretion is the most common function of PCATs and that five of the seven *rtg* GG peptide genes are always associated with downstream genes encoding hypothetical proteins that resemble bacteriocin immunity proteins (43). The fact that the Sp9-BS68 strain with a functional *rtg* locus demonstrated a competitive advantage over the Δ*rtgR* Δ*rtgS1* strain during dual infection is consistent with the bacteriocin hypothesis, as the regulator mutant would be unable to upregulate immunity, although we cannot exclude that other *rtgR*-regulated factors play a role in this fitness advantage. Regardless of the specific function of the *rtg* GG peptides, the fact that most RtgAB^−^ strains are still RtgR^+^ suggests that *rtg* retains a useful function that does not require secretion of these peptides. Further studies will be needed to determine the mechanism responsible for the RtgR/S-dependent competitive fitness advantage seen in colonization studies, the function of the *rtg* GG peptides, and the biological significance of active *rtg* loci with nonfunctional RtgAB.

The case of RtgAB and ComAB/BlpAB allowed us to study how two sets of PCATs which coexist in the same strain preferentially secrete different sets of peptides through slight differences in substrate recognition. Unlike ComAB and BlpAB, RtgAB recognizes its substrates partially using a motif located at the N-terminal ends of their signal sequences. This motif is located 18 residues away from the signal sequence cleavage site and is exclusively found in *rtg* GG peptides. Where data are available, previous studies of PCAT substrates have found that positions at the N terminus located farther than 18 residues from the cleavage site are either dispensable for recognition by PCATs (28, 30) or can be missing entirely (33, 44, 45). As far as we are aware, RtgAB is unique among PCATs in recognizing a signal sequence motif located so distantly from the cleavage site. Future efforts will be directed toward identifying the specific nature of the interaction between the N-terminal motif and RtgAB and the exact signal sequence residues involved.

The insights into the sequence determinants of PCAT substrate selectivity gained here illuminate a relatively understudied aspect of this class of transporters. They will also be useful in guiding future efforts to predict substrates for ComAB, BlpAB, RtgAB, and other PCATs. Some GG peptides are found without a closely associated or coregulated PCAT (18). In these cases, it would be helpful to have sequence-based approaches for assigning potential transporters to these “orphan” GG peptides. Moreover, for strains that harbor multiple PCATs, predicting if GG peptides can be secreted by PCATs that are not necessarily closely associated can guide mechanistic studies that lead to new insights into function and regulation, such as with ComAB and BlpAB substrates in pneumococcus. Our work lays the groundwork for identifying signal sequence motifs of GG peptides that are important for transporter selectivity. The next step will be to study the corresponding sequence and structural motifs in PCATs that contribute to this selectivity. In addition to bacteriocins and quorum sensing (3), GG peptides have now been linked to biofilm formation, colonization of host niches, and dissemination during infection (18, 46). Ultimately, the ability to predict and rationalize PCAT-GG peptide pairings will advance our understanding of a broad range of biologically significant microbial processes.

## MATERIALS AND METHODS

### Strains and growth conditions.

All strains were derived from Sp9-BS68 (36), D39, or the R6 strain P654 (referred to as PSD100 in reference 47) (see Table S1 and methods in Text S1 in the supplemental material for details). The modified R6 strain was used for some *in vitro* assays because previous work demonstrating the *blp-com* connection was performed in this strain background. Pneumococcus was grown in either filter-sterilized THY (Todd Hewitt broth plus 0.5% yeast extract) or CDM+ (see methods in Text S1) (38) at 37°C. All media contained 5 μg/ml catalase. All CDM+ was supplemented with 0.5% (vol/vol) THY. Except where noted otherwise, pneumococcal cultures used for experiments were inoculated to an OD_620_ of 0.0015 from starter cultures grown in THY (pH 7.4) to an OD_620_ of 0.275 and frozen at −80°C in 13% glycerol. Starter cultures were pelleted at 6,000 × *g* for 5 min at room temperature and resuspended in the appropriate growth medium for the experiment before being used for inoculation. Antibiotics were used at the following concentrations: chloramphenicol, 2 μg/ml; gentamicin, 200 μg/ml; kanamycin, 500 μg/ml; spectinomycin, 200 μg/ml; streptomycin, 100 μg/ml.

10.1128/mBio.02502-19.8 TABLE S1 Strains and primers used in this work. Download Table S1, PDF file, 0.2 MB. Copyright © 2020 Wang et al. 2020 Wang et al. This content is distributed under the terms of the Creative Commons Attribution 4.0 International license.

10.1128/mBio.02502-19.9 TEXT S1 Supplemental methods. Download Text S1, PDF file, 0.3 MB. Copyright © 2020 Wang et al. 2020 Wang et al. This content is distributed under the terms of the Creative Commons Attribution 4.0 International license.

### Transformations.

Transformation protocols were adapted from those described in reference 48. See methods in Text S1 and Table S1 for details and primers used for constructing transforming DNA products. Unmarked chromosomal mutations were created via Janus (49), Sweet Janus (50), or Janus2 (Text S1) exchange. Transformants were verified by Sanger sequencing.

### Luciferase reporter time course assays.

For *com*-*blp* activation assays only, starter cultures were grown in THY (pH 6.8) to an OD_620_ of 0.075 to prevent *com*-*blp* activation. Cells were grown in THY or CDM+ in a white, clear-bottom 96-well plate (655098; Greiner Bio-One), 200 μl per well. For assays using firefly luciferase, the following concentrations of firefly luciferin (88294; Thermo Fisher Scientific) were added to the medium: 330 μM (single reporter and dual reporter, CDM+), 165 μM (dual reporter, THY). For assays using NanoLuc luciferase, the following concentrations of Nano-Glo substrate (N1121; Promega) were added to the media: 1:5,000 (CDM+), 1:10,000 (THY). The plate was incubated in a Synergy HTX plate reader set to read absorbance at 620 nm and luminescence every 5 min. For single reporter assays, no filter was used for luminescence readings. For dual reporter assays, 450/50 band-pass and 610 long-pass filters were used to isolate NanoLuc and red firefly luciferase signals, respectively. For D39 strains only, the plate was shaken before readings were taken. Promoter activities were calculated from luminescence and absorbance readings as described in reference 25. For locus activation assays, timings of activation events were calculated as described in the methods in Text S1 and compared using survival analysis. Differences between groups were assessed by log-rank tests using the FHtest package (v1.4) in R, and when appropriate, the Holm correction was applied for multiple comparisons.

### RtgS dose-response assays.

Cells expressing P*_rtgS1_*-*luc* reporters were grown in THY or CDM+ containing 330 μM firefly luciferin. At an OD_620_ of 0.02, cultures were aliquoted into a white, clear-bottom 96-well plate (655098; Greiner Bio-One), 100 μl per well. Each well of the plate was prefilled with 100 μl sterile medium containing 0.5% (vol/vol) dimethyl sulfoxide (DMSO), 330 μM firefly luciferin, and appropriate concentrations of synthetic RtgS peptide (Genscript). The plate was then incubated in a Synergy HTX plate reader set to read absorbance at 620 nm and luminescence every 5 min. For D39 strains only, the plate was shaken before readings were taken. P*_rtgS1_* activity was calculated, and the response was defined as the maximum observed P*_rtgS1_* activity within 60 min (Sp9-BS68) or 120 min (D39) of treatment. When applicable, curves were fit to a Hill model using the nls() function in R 3.5.1.

### Peptide secretion assays.

Cells were inoculated from starter cultures to an OD_620_ of 0.005 and grown in CDM+. At an OD_620_ of 0.05, cells were treated with 200 ng/ml CSP1, 200 ng/ml BlpC_R6_, and 20 nM RtgS_A_-C10. Samples were taken for HiBiT quantification at appropriate time points. For native BlpI-HiBiT assays only, clarified supernatants were obtained after centrifugation at 6,000 × *g* for 5 min at 4°C. For all other assays, cells were retained in the samples. HiBiT signal was quantified by mixing samples with HiBiT extracellular detection reagent (N2421; Promega) at a 1:1 ratio and reading luminescence with a Synergy HTX plate reader. Samples were also taken for quantification of intracellular peptide; for endpoint assays, they were taken concurrently with the extracellular samples, and for time course assays, they were taken at the last time point. Extracellular peptide was removed from these samples by proteinase K digestion, and then the cells were lysed and HiBiT signal was quantified as described above. Standards consisting of synthetic L10-HiBiT peptide (25) mixed with samples of a non-HiBiT-expressing strain were used to generate standard curves to use for calculating HiBiT-tagged peptide concentrations in experimental samples. See methods in Text S1 for more details. Differences between groups were assessed by analysis of variance (ANOVA) using the emmeans package (v1.2.3) in R.

### Genomic analysis of *rtg*.

Analysis of *rtg* was performed using the assembled genomes of the Massachusetts isolate collection (BioProject accession PRJEB2632). See methods in Text S1 for details.

### Mouse colonization assays.

Mouse colonization was performed as described in reference 25. Briefly, dual or single-strain mixtures of Sp9-BS68 were inoculated into the nasopharynx of unanaesthetized 5- to 7-week-old female BALB/c mice (Taconic)with 1.0 × 10^6^ to 3.0 × 10^6^ CFU/mouse in 10 μl of sterile phosphate-buffered saline (PBS). For dual inoculated mice, the ratio of the kanamycin-resistant strain to the spectinomycin-resistant strain was between 0.25 and 0.6. Mice were euthanized with CO_2_ overdose after 72 h, and nasopharyngeal colonization was sampled by nasal wash. See methods in Text S1 for IACUC approval and details on how colonization density and competitive indices were calculated. Differences in colonization densities and competitive indices between groups were evaluated by the Mann-Whitney (2 groups) and Kruskal-Wallis (>2 groups) tests using the wilcox.test() and kruskal.test() functions in R 3.5.1.

### Data availability.

Sequences of Janus+ and Janus2 constructs were deposited in GenBank under accession numbers MN848328 and MN848329, respectively. The *rtg* locus from Sp9-BS68 including the new sequencing that allowed us to connect existing contigs and *rtg* gene designations established here was deposited as accession number MN848330.
