# Supplementary figures and images for: Molecular Determinants of Substrate Selectivity of a Pneumococcal Rgg-Regulated Peptidase-Containing ABC Transporter
Source: mBio. 2020 Feb 11;11(1):e02502-19. doi: 10.1128/mBio.02502-19 (PMC7018657; doi:10.1128/mBio.02502-19)

**A**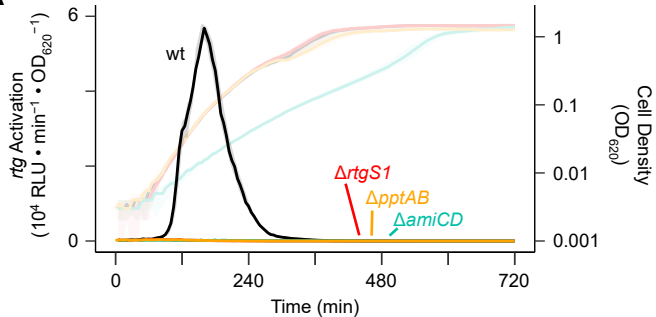**B**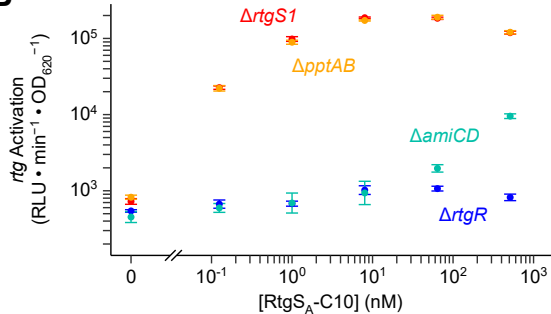

Supplement: FIG S1 [file mBio.02502-19-sf001.pdf]

*rtqR*

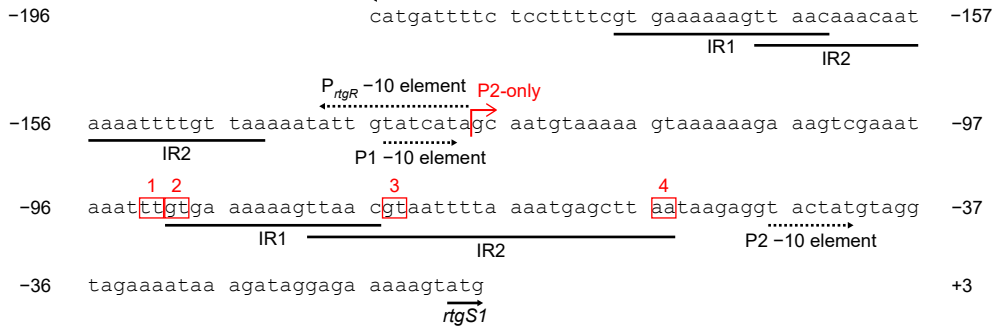

# B

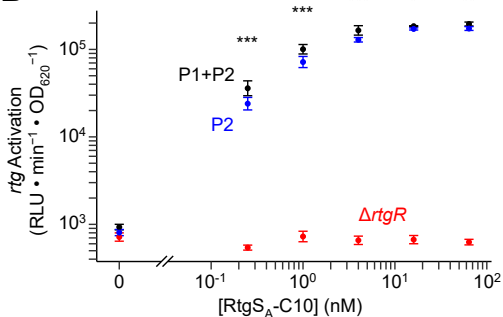

**C**

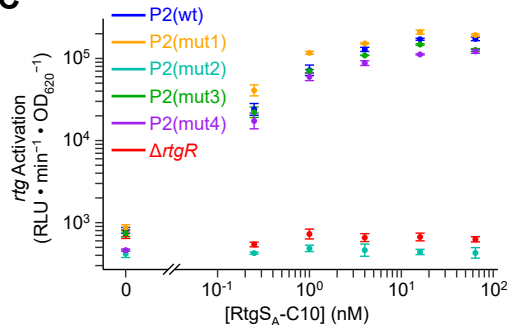

Supplement: FIG S2 [file mBio.02502-19-sf002.pdf]

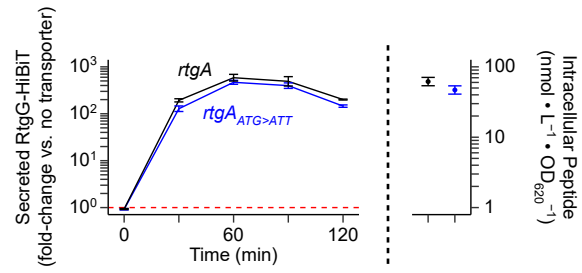

Supplement: FIG S4 [file mBio.02502-19-sf004.pdf]

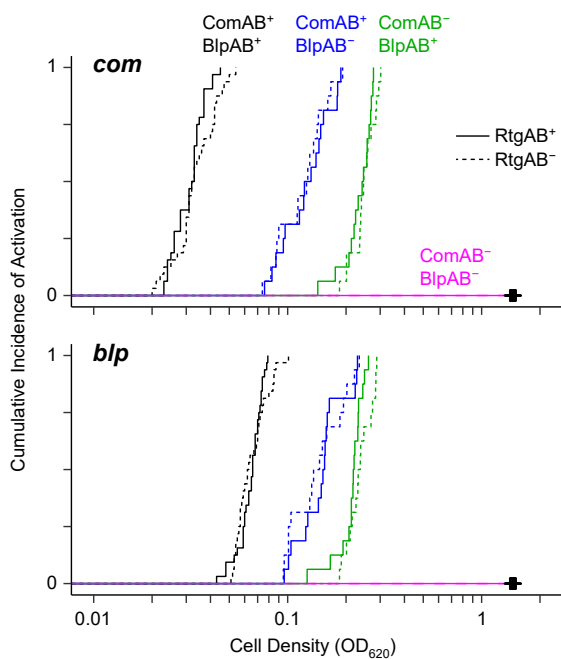

Supplement: FIG S5 [file mBio.02502-19-sf005.pdf]

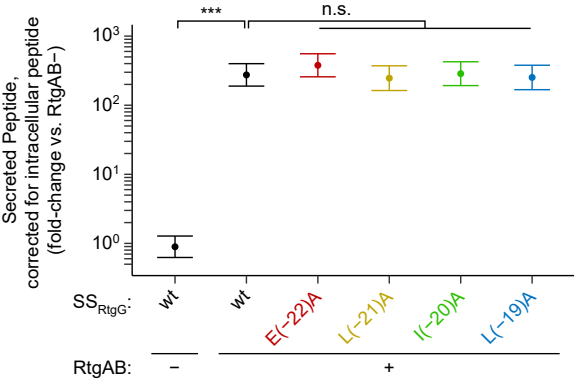

Supplement: FIG S6 [file mBio.02502-19-sf006.pdf]

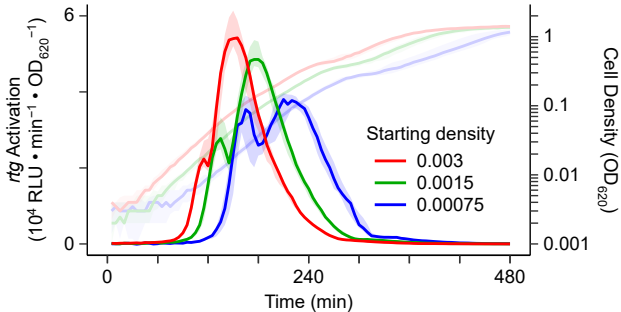

Supplement: FIG S7 [file mBio.02502-19-sf007.pdf]
