# Supplementary material for: Molecular Determinants of Substrate Selectivity of a Pneumococcal Rgg-Regulated Peptidase-Containing ABC Transporter
Source: mBio. 2020 Feb 11;11(1):e02502-19. doi: 10.1128/mBio.02502-19 (PMC7018657; doi:10.1128/mBio.02502-19)
Supplement: FIG S3 [file mBio.02502-19-sf003.pdf]

A

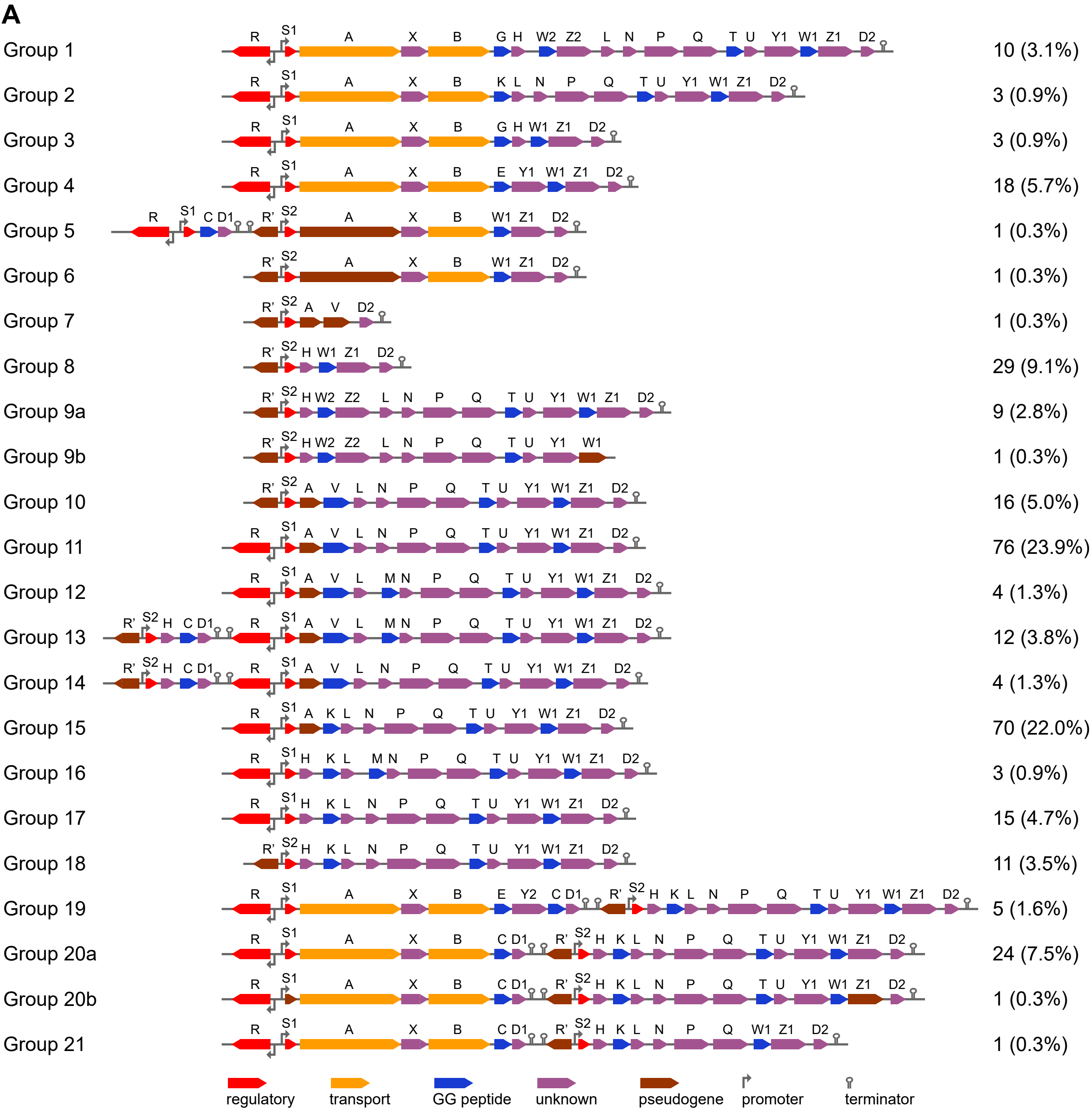

B

Peptide	*Signal Sequence*	*Strain*	*Group*
RtgC*	M E L V L P N N Y V D L E Q E E M M Y L D **G G**	D39	20a
RtgE	M E L V L P N N Y V V I D E E E M M Y L D **G G**	101058	4
RtgG*	M E L I L P N N Y V D L E Q E E M M Y L D **G G**	Sp9-BS68	1
RtgK	M E L V L P N N Y V V I D E E E M M Y L D **G G**	D39	20a
RtgM	M E L V L P N N Y V V L E Q E E M I Y L D **G G**	503574	12
RtgT	M E L V L P N N Y V V L E Q E E M M Y L D **G G**	D39	20a
RtgV	M E L V L P N N Y V A L E Q E E M M Y L D **G G**	LE4038	11
RtgW1	M E L V L P N N Y V V I D E E E M M Y L D **G G**	Sp9-BS68	1
RtgW2	M E L V L P N N Y V A L E Q E E M M Y L D **G G**	Sp9-BS68	1

−20 −15 −10 −5 −1
