# Supplementary material for: Molecular Determinants of Substrate Selectivity of a Pneumococcal Rgg-Regulated Peptidase-Containing ABC Transporter
Source: mBio. 2020 Feb 11;11(1):e02502-19. doi: 10.1128/mBio.02502-19 (PMC7018657; doi:10.1128/mBio.02502-19)
Supplement: TABLE S1 [file mBio.02502-19-st001.pdf]

Table S1. Strain and Primer list.

Strain or Primer	*Description or sequence*	*Antibiotic Resistances*	*Reference*
Sp9-BS68	serotype 9 clinical isolate	None	1
P2055	D39, *rpsL*$_{167A>C}$, Δ*blpT-blpA*::[*blpT-blpA*]$_{P32}$, ΔBIR::BIR$_{P164}$	str^R	2
P2538	R6, *rpsL*$_{167A>C}$, Δ*blpA*::*blpA*$_{P654,468\_469insAAGC}$, Δ*blpB*::*blpB*$_{P654}$, ΔBIR::BIR$_{P654}$, pE57 insertion in *blpA-blpQ* region (P$_{BIR}$-*lacZ*), Δ*comAB*, Δ*blpC*::*blpC*$_{6A}$, Δ*comC*, Δ*blpQ-pncT*::*blpI*$_{P133}$-*strepII*	cam^R, str^R	2
P2565	R6, *rpsL*$_{167A>C}$, Δ*blpA*::*blpA*$_{P654,468\_469insAAGC}$, Δ*blpB*::*blpB*$_{P654}$, ΔBIR::BIR$_{P654}$, pE57 insertion in *blpA-blpQ* region (P$_{BIR}$-*lacZ*), Δ*comAB*, Δ*blpC*::*blpC*$_{6A}$, Δ*comC*::*comC*$_{TIGR4}$, Δ*blpQ-pncT*::*blpI*$_{P133}$-*HiBiT*	cam^R, str^R	2
P2567	R6, *rpsL*$_{167A>C}$, Δ*blpA*::*blpA*$_{P654,468\_469insAAGC}$, Δ*blpB*::*blpB*$_{P654}$, ΔBIR::BIR$_{P654}$, pE57 insertion in *blpA-blpQ* region (P$_{BIR}$-*lacZ*), Δ*blpC*::*blpC*$_{6A}$, Δ*comC*::*comC*$_{TIGR4}$, Δ*blpQ-pncT*::*blpI*$_{P133}$-*HiBiT*	cam^R, str^R	2
P2569	R6, *rpsL*$_{167A>C}$, Δ*blpAB*::*blpAB*$_{P654}$, ΔBIR::BIR$_{P654}$, pE57 insertion in *blpA-blpQ* region (P$_{BIR}$-*lacZ*), Δ*comAB*, Δ*blpC*::*blpC*$_{6A}$, Δ*comC*::*comC*$_{TIGR4}$, Δ*blpQ-pncT*::*blpI*$_{P133}$-*HiBiT*	cam^R, str^R	2
P2665	D39, *rpsL*$_{167A>C}$, Δ*blpAB*::*blpAB*$_{P654}$, ΔBIR::BIR$_{P164}$, pE57 insertion in *blpA-blpI* region with Δ*lacZ*::*RFluc* (P$_{BIR}$-*RFluc*), Δ*bgaA*::P$_{comA}$-*Nluc*	cam^R, str^R	2
P2666	P2665 with Δ*blpA*::*blpA*$_{468\_469insAAGC}$	cam^R, str^R	2
P2668	P2665 with Δ*comAB*	cam^R, str^R	2
P2670	P2665 with Δ*blpA*::*blpA*$_{468\_469insAAGC}$, Δ*comAB*	cam^R, str^R	2
P2772	Sp9-BS68 with CEP-PF6-*luc*	None	This study
P2775	Sp9-BS68 with CEP-P$_{rtgA}$-*luc*	None	This study
P2790	P2055 with CEP-P$_{rtgS1}$-*luc*	str^R	This study
P2792	Sp9-BS68 with CEP-P$_{rtgS1}$-*luc*	None	This study
P2802	P2792 with Δ*rtgR*	None	This study
P2804	P2792 with Δ*rtgS1*	None	This study
P2811	P2792 with Δ*rtgR*, Δ*rtgS1*	None	This study
P2838	P2665 with Δ*rtgAXB*::*rtgAXB*$_{Sp9-BS68}$	cam^R, str^R	This study
P2840	P2666 with Δ*rtgAXB*::*rtgAXB*$_{Sp9-BS68}$	cam^R, str^R	This study
P2842	P2668 with Δ*rtgAXB*::*rtgAXB*$_{Sp9-BS68}$	cam^R, str^R	This study
P2844	P2670 with Δ*rtgAXB*::*rtgAXB*$_{Sp9-BS68}$	cam^R, str^R	This study
P2859	P2790 with Δ*rtgS1*	str^R	This study
P2878	P2538 with Δ*rtgS1*, Δ*rtgC-rtgD2*	cam^R, str^R	This study
P2880	P2565 with Δ*rtgS1*, Δ*rtgC-rtgD2*	cam^R, str^R	This study
P2882	P2565 with Δ*rtgS1*, Δ*rtgAXB*::*rtgAXB*$_{Sp9-BS68}$, Δ*rtgC-rtgD2*	cam^R, str^R	This study
P2884	P2567 with Δ*rtgS1*, Δ*rtgC-rtgD2*	cam^R, str^R	This study
P2888	P2569 with Δ*rtgS1*, Δ*rtgC-rtgD2*	cam^R, str^R	This study
P2908	P2790 with Δ*rtgS2*	str^R	This study
P2910	P2790 with Δ*rtgS1*Δ*rtgS2*	str^R	This study
P2934	P2880 with ΔpE57, Δ*blpI*$_{P133}$-*HiBiT*::*blpQ-pncT*	str^R	This study
P2936	P2882 with ΔpE57, Δ*blpI*$_{P133}$-*HiBiT*::*blpQ-pncT*	str^R	This study
P2938	P2884 with ΔpE57, Δ*blpI*$_{P133}$-*HiBiT*::*blpQ-pncT*	str^R	This study
P2940	P2888 with ΔpE57, Δ*blpI*$_{P133}$-*HiBiT*::*blpQ-pncT*	str^R	This study
P2959	P2934 with *rtgB*-[*rtgC-HiBiT*]-*rtgD1*	str^R	This study
P2961	P2936 with *rtgB*-[*rtgC-HiBiT*]-*rtgD1*	str^R	This study
P2963	P2938 with *rtgB*-[*rtgC-HiBiT*]-*rtgD1*	str^R	This study
P2965	P2940 with *rtgB*-[*rtgC-HiBiT*]-*rtgD1*	str^R	This study
P2967	P2934 with *rtgB*-[*rtgG-HiBiT*]-*rtgH*	str^R	This study
P2969	P2936 with *rtgB*-[*rtgG-HiBiT*]-*rtgH*	str^R	This study
P2971	P2938 with *rtgB*-[*rtgG-HiBiT*]-*rtgH*	str^R	This study

Strain or Primer	*Description or sequence*	*Antibiotic Resistances*	*Reference*
P2973	P2940 with *rtgB*-[*rtgG-HiBiT*]-*rtgH*	str^R	This study
P2981	P2934 with *rtgB*-[SS_{rtgG}*-blpI-HiBiT*]-*rtgH*	str^R	This study
P2983	P2936 with *rtgB*-[SS_{rtgG}*-blpI-HiBiT*]-*rtgH*	str^R	This study
P2985	P2938 with *rtgB*-[SS_{rtgG}*-blpI-HiBiT*]-*rtgH*	str^R	This study
P2987	P2940 with *rtgB*-[SS_{rtgG}*-blpI-HiBiT*]-*rtgH*	str^R	This study
P2993	P2934 with *rtgB*-[SS_{blpI}*-rtgG-HiBiT*]-*rtgH*	str^R	This study
P2995	P2936 with *rtgB*-[SS_{blpI}*-rtgG-HiBiT*]-*rtgH*	str^R	This study
P2997	P2938 with *rtgB*-[SS_{blpI}*-rtgG-HiBiT*]-*rtgH*	str^R	This study
P2999	P2940 with *rtgB*-[SS_{blpI}*-rtgG-HiBiT*]-*rtgH*	str^R	This study
P3001	P2792 with CEP-*spcR*	spc^R	This study
P3003	P2811 with CEP-*spcR*	spc^R	This study
P3009	P2934 with *rtgB*-[SS_{rtgG}*(F/M/L/V)-blpI-HiBiT*]-*rtgH*	str^R	This study
P3011	P2936 with *rtgB*-[SS_{rtgG}*(F/M/L/V)-blpI-HiBiT*]-*rtgH*	str^R	This study
P3013	P2938 with *rtgB*-[SS_{rtgG}*(F/M/L/V)-blpI-HiBiT*]-*rtgH*	str^R	This study
P3015	P2940 with *rtgB*-[SS_{rtgG}*(F/M/L/V)-blpI-HiBiT*]-*rtgH*	str^R	This study
P3017	P2934 with *rtgB*-[SS_{blpI}*(Y/L/M/L)-blpI-HiBiT*]-*rtgH*	str^R	This study
P3019	P2936 with *rtgB*-[SS_{blpI}*(Y/L/M/L)-blpI-HiBiT*]-*rtgH*	str^R	This study
P3021	P2938 with *rtgB*-[SS_{blpI}*(Y/L/M/L)-blpI-HiBiT*]-*rtgH*	str^R	This study
P3023	P2940 with *rtgB*-[SS_{blpI}*(Y/L/M/L)-blpI-HiBiT*]-*rtgH*	str^R	This study
P3025	P2811 with CEP-*genR*	gen^R	This study
P3027	P2934 with *rtgB*-[*blpI-HiBiT*]-*rtgH*	str^R	This study
P3029	P2936 with *rtgB*-[*blpI-HiBiT*]-*rtgH*	str^R	This study
P3031	P2938 with *rtgB*-[*blpI-HiBiT*]-*rtgH*	str^R	This study
P3033	P2940 with *rtgB*-[*blpI-HiBiT*]-*rtgH*	str^R	This study
P3035	P3001, mouse passaged	spc^R	This study
P3037	P3003, mouse passaged	spc^R	This study
P3039	P3025, mouse passaged	gen^R	This study
P3055	P2934 with *rtgB*-[$SS_{rtgG}(N6_{blpI})$*-blpI-HiBiT*]-*rtgH*	str^R	This study
P3057	P2936 with *rtgB*-[$SS_{rtgG}(N6_{blpI})$*-blpI-HiBiT*]-*rtgH*	str^R	This study
P3059	P2938 with *rtgB*-[$SS_{rtgG}(N6_{blpI})$*-blpI-HiBiT*]-*rtgH*	str^R	This study
P3061	P2940 with *rtgB*-[$SS_{rtgG}(N6_{blpI})$*-blpI-HiBiT*]-*rtgH*	str^R	This study
P3075	P2792 with Δ*amiCD*	none	This study
P3077	P2792 with Δ*pptAB*	none	This study
P3081	P2934 with *rtgB*-[$SS_{blpI}(N6_{rtgG})$*-blpI-HiBiT*]-*rtgH*	str^R	This study
P3083	P2936 with *rtgB*-[$SS_{blpI}(N6_{rtgG})$*-blpI-HiBiT*]-*rtgH*	str^R	This study
P3085	P2938 with *rtgB*-[$SS_{blpI}(N6_{rtgG})$*-blpI-HiBiT*]-*rtgH*	str^R	This study
P3087	P2940 with *rtgB*-[$SS_{blpI}(N6_{rtgG})$*-blpI-HiBiT*]-*rtgH*	str^R	This study
P3100	P2804 with CEP-P_{rtgS1}(P2)-*luc*	none	This study
P3123	P3100 with CEP-P_{rtgS1}(P2-[−82_−81TT>GG])-*luc*	none	This study
P3125	P3100 with CEP-P_{rtgS1}(P2-[−80_−79GT>TG])-*luc*	none	This study
P3127	P3100 with CEP-P_{rtgS1}(P2-[−65_−64GT>TG])-*luc*	none	This study
P3129	P3100 with CEP-P_{rtgS1}(P2-[−46_−45AA>CC)-*luc*	none	This study
P3111	P2934 with *rtgB*-[SS_{rtgG}*(A/A/A/A)-blpI-HiBiT*]-*rtgH*	str^R	This study
P3113	P2936 with *rtgB*-[SS_{rtgG}*(A/A/A/A)-blpI-HiBiT*]-*rtgH*	str^R	This study
P3133	P2936 with *rtgB*-[SS_{rtgG}*[E(−22)A]-blpI-HiBiT*]-*rtgH*	str^R	This study
P3135	P2936 with *rtgB*-[SS_{rtgG}*[L(−21)A]-blpI-HiBiT*]-*rtgH*	str^R	This study
P3137	P2936 with *rtgB*-[SS_{rtgG}*[I(*−20*)A]-blpI-HiBiT*]-*rtgH*	str^R	This study
P3138	P2936 with *rtgB*-[SS_{rtgG}*[L(*−19*)A]-blpI-HiBiT*]-*rtgH*	str^R	This study
P3142	P2934 with *rtgB*-[SS_{blpI}*(A/A/A/A)-blpI-HiBiT*]-*rtgH*	str^R	This study
P3144	P2938 with *rtgB*-[SS_{blpI}*(A/A/A/A)-blpI-HiBiT*]-*rtgH*	str^R	This study
P3146	P2940 with *rtgB*-[SS_{blpI}*(A/A/A/A)-blpI-HiBiT*]-*rtgH*	str^R	This study
P3160	P2936 with *rtgB*-[SS_{rtgG}*[P(−18)M]-blpI-HiBiT*]-*rtgH*	str^R	This study
P3162	P2969 with Δ*rtgA*::$rtgA_{ATG>ATT}$	str^R	This study
P3166	P2936 with *rtgB*-[SS_{rtgG}*[ΔN(2-6)]-blpI-HiBiT*]-*rtgH*	str^R	This study

Strain or Primer	*Description or sequence*	*Antibiotic Resistances*	*Reference*
P3170	P2936 with *rtgB*-[SS_{blpI}*[ΔN(2-6)]-blpI-HiBiT*]-*rtgH*	str^R	This study

Primer	*Sequence (5' to 3')*
CW105	TCCGTTTGATTTTTAATGGATAATGTG
CW106	CAGAGACCTGGGCCCCTTTC
CW158	ATGGAAGACGCCAAAAACATAAAG
CW188	GGAACAACTTTACCGACCGC
CW189	CCCGGTATCCAGATCCACAA
CW190	GGGCGTATCTCTTCATAGCCT
CW191	GAAGAGATACGCCCTGGTTCC
CW219	TCAATCAGGACAGTCAAATCGA
CW234	TCTTCAAAAGTCGTGCGTTGA
CW238	cattaaaaatcaaacggaTCTTTTGCAAACGAGTCGCT
CW270	cccaggtctctgTGAACCAACAAACGACTTTTAGT
CW271	AGGTATGTAGGCGGTGCTAC
CW272	AACCCGGTAAGACACGACTT
CW292	TGGAATTGACTCGATAGCTTTAACA
CW293	AAAACAAACCGCATCCGTGT
CW294	TCCAACCTAACCAGCTACCA
CW295	CTGCGCTCTAAAACCAACGT
CW303	TCTGTACGTGCCCAAGTCTT
CW343	TGAAAGCTGGCGCATGATG
CW357	GATATCCTCCCTGATCGACCG
CW358	ATCAGGGAGGATATCGGGGA
CW375	acttttcctccTTCTTTTGCGTTTTTATTTGTTAACTGT
CW380	TAATAGAACGCATGAGAAAGCCC
CW406	CGAGTTACTGGAGGGATCCTTA
CW406	CGAGTTACTGGAGGGATCCTTA
CW407	agaaggaggaaaagtATGTTACGCAGCAGCAACG
CW446	AATACCACTGACAGTCGTTCCA
CW447	ACTGTCAGTGGTATTGTTTCAGG
CW451	AGAAGGTTTACAAGCGAGCTC
CW454	cattaaaaatcaaacggaCCACCTTTTTCATCACCTGTCAA
CW455	ctcatgcgttctattaCCACCTTTTTCATCACCTGTCAA
CW456	AGCACTTGACTCGTTACCCT
CW457	ctccagtaactcgATGGAAGACGCCAAAAACATAAAG
CW458	cgccagctttcaTTACAATTTGGACTTTCCGCCC
CW463	AGTAAGAAGTTTGTCGCGGTT
CW464	TGGCTGACTAGGAGGAAGGA
CW468	ACGATACCAAACTTGTCTGCAA
CW471	cattaaaaatcaaacggaGGAAGCGACCAATAATCTCATCA
CW474	GTTAGACACTAAAAGAATCTTGCTTGG
CW481	ttcttttagtgtctaacCCCACTACAAATATTATAGAGCCGA

Primer	*Sequence (5' to 3')*
CW482	ctcatgcgttctattaGGAAGCGACCAATAATCTCATCA
CW483	ACCTACATAGTACCTCTTATTAAGCTCA
CW484	GAGGTACTATGTAGGTAGAAAATAAAGATAGG
CW485	TGAGGTTCAAAAAAGCGCCA
CW486	GACTTTCCCCATTGATCGCC
CW487	CACCTCGTCCATGATAACTTTTGA
CW488	CGAGATAGAAACAAAAGAACGTGC
CW489	ctccagtaactcgCTCTGCCAAGCCACATAGTG
CW490	cattaaaaatcaaacggaAGCAATGTAAAAAGTAAAAAAGAAAGTCGA
CW491	tagatctcTGACTCACGTATTTCTTTATAGACCTTT
CW492	tgagtcaGAGATCTATTACCAAGCGAAGGAAAG
CW494	tggcgtcttcCATACTTTTTCTCCTATCTTTATTTTCTACCTAC
CW496	TCCTCGGTTAGCTCCAGAAG
CW498	GGACAACCGCACTAATAACTGA
CW499	AGTCCATTTGCTTCCATTTGTTCA
CW500	cattaaaaatcaaacggaACCTACATAGTACCTCTTATTAAGCTCA
CW501	ctccagtaactcgGGCAGTGATTGAGGTTTGGG
CW502	CTCAATCACTGCCCAGTAGTTC
CW503	GGCAGTGATTGAGGTTTGGG
CW506	CAGCCCAATCAGCCCCATAT
CW509	cccaggtctctgGGAAATGTTAAGTTTCAAGCAATAATTGG
CW511	CTTGAAACTTAACATTTCTAAAATCCAATCAAG
CW513	CACTATGTGGCTTGGCAGAG
CW518	AGAATCTAATGACCGAGCTACTGT
CW519	ACACCTAAATAAAGTCATCTCTCCAA
CW520	GTGATCGCAGGCTTTGTTTAGT
CW521	GCCTGCGATCACTTGTTAGG
CW522	GAAATGTTAAGTTTCAAGCAATAATTGG
CW523	cattaaaaatcaaacggaGGCAGTGATTGAGGTTTGGG
CW531	CAGCAATTAGTTCAGGGAAGGT
CW534	cattaaaaatcaaacggaACAATCCGCACTTCAACTGG
CW537	cattaaaaatcaaacggaCCCAAACCTCAATCACTGCC
CW538	cccaggtctctgGCACAACATAGGGAGTTAGAGAAG
CW539	CTCCGCTTTTGTCTCTTCGT
CW541	agtccaaactattctTTATTGCTTGAAACTTAACATTTCTAAAATCC
CW542	AGAATAGTTTGGACTTGAACTGTATCTA
CW543	GCAATTGGTTCAGGGAAGGT
CW545	GGTACTGCTCGTTCAAACAACA
CW546	cattaaaaatcaaacggaTTATTGCTTGAAACTTAACATTTCTAAAATCC
CW548	TTGCTCGAATTTATAGGACTTTTTTCTAT
CW550	GCAACAAAACCAGCCCCTAA
CW551	GCGGCTTTCTCCTCAGTTTT
CW556	AGACTTCTAGGCGTTGCAATATT
CW557	ATAGAAAAAAGTCCTATAAATTCGAGCAA

Primer	*Sequence (5' to 3')*
CW559	CGTTTATCCCAACTCAATTATGACATT
CW561	cattaaaaatcaaacggaTGAGTTGTTAGTGGAGGATAAAATGT
CW562	cccaggtctctgACCTACATAGTACCTCTTATTAAGCTCA
CW565	agtccaaactattctTTATTGCTTGAAACTTAACATTTCCAAAA
CW568	ACAAGTGATCCTCGACTGTGT
CW570	ACAATTTTCGAAAAAACCCGCTTC
CW571	gttttttcgaaaattgtCCCCCGTTTGATTTTTAATGGATAAT
CW572	cgccagctttcaCGCCCTTCAATTTTTTTATAATTTTTTTAATCT
CW584	GACCATTCTTTTTATCTTTATGGGGTT
CW585	TGGGTTTGGGAGCTAGAAAAATAG
CW586	ATCGAAACGTCTTGAATTAGCTTTTTTA
CW587	TCAAGACGTTTCGATGCCAA
CW592	GCTGATTGGGCTGACGGTA
CW593	TAGATACAGTTCAAGTCCAAACTATTCT
CW594	GACTTGAACTGTATCTAAAAACAGAGG
CW595	CTTGAAACTTAACATTTCCAAAATCCAATC
CW596	CCAACCAGAAACAGAACCTCC
CW597	CTGTTTCTGGTTGGCGTCTT
CW598	ATGTTACGCAGCAGCAACG
CW599	cgccagctttcaTTAGGTGGCGGTACTTGGG
CW600	tgctgcgtaacatATATATCCTCCTCACTATTTTGATTAGTACC
CW606	TTTCCCACTACCGGACTCAC
CW607	CCTTCCTCCTAGTCAGCCAG
CW611	cattaaaaatcaaacggaCGGCGTTTCCCTTGAACTAG
CW612	taccttgTCCCTTTTCGTAGTTGGTCAAAA
CW613	aaaagggaCAAGGTATAGATAATCGTGTAGGTCAA
CW614	ctccagtaactcgCCCAAAAGAACCTTCTCCTCATG
CW616	GCCTTGAATTTTGCTCTTGATCG
CW617	TGCTGGTCTGGGTGTGAATA
CW618	GAGTTACCTAAATTATGATGCATAGTTGA
CW619	GAGGATTTTAAAGTAATCTCTAACAATGCT
CW622	cattaaaaatcaaacggaAGGAACATGAACATAGCCACCT
CW623	aaccagcAGGAACATGAACATAGCCACCT
CW624	gttcctGCTGGTTTGGTTTTACTAGTCTTG
CW625	ctccagtaactcgCATTGCTGATACGACACTAAAAAAGA
CW627	GGCATACTTGACAGCGTATTGA
CW628	AGTTTCTCAATCGTCGTTTCAATTC
CW629	gttttttcgaaaattgtGCAATGTAAAAAGTAAAAAAGAAAGTCGA
CW630	cttttttccaaaATTTATTTCGACTTTCTTTTTTACTTTTTACATTG
CW631	taaattttgGAAAAAAGTTAACGTAATTTTAAAATGAGCTT
CW632	ctttttttcacccATTTATTTCGACTTTCTTTTTTACTTTTTACATTG
CW633	taaatgggtGAAAAAAGTTAACGTAATTTTAAAATGAGCTT
CW634	aaaattcagtTAACTTTTTTCACAAATTTATTTCGACTTTCT
CW635	aaagttaactgAATTTTAAAATGAGCTTAATAAGAGGTACTATGT

Primer	*Sequence (5' to 3')*
CW636	cctcttaggAAGCTCATTTTAAAATTACGTTAACTTTTTTC
CW637	agcttcctaaGAGGTACTATGTAGGTAGAAAATAAAGATAGG
CW640	GTGGGGTATTAGGGGAGCAA
CW649	tcaagatcaacataattattcatCAATATGAGTTCCATTTATTTTCTCCTTTT
CW651	AATAATTATGTTGATCTTGAACAAGAAGAGA
CW652	caacataattatttggagcTATGAGTTCCATTTATTTTCTCCTTTTTTATTAAG
CW653	caacataattatttggcaatgcGAGTTCCATTTATTTTCTCCTTTTTTATTAAGC
CW654	caacataattatttggcaatatagcTTCCATTTATTTTCTCCTTTTTTATTAAGCAT
CW655	caacataattatttggcaatatgagtgcCATTTATTTTCTCCTTTTTTATTAAGCATGGT
CW656	CCAAATAATTATGTTGATCTTGAACAAGAAG
CW657	ctccagtaactcgTCAAAAGTTATCATGGACGAGGTG
CW659	CGAATTTTCCCCTCACTCGG
CW660	TGTCCCTGTAAAGAGTTCTATGGT
CW665	TGATACAGGAATGCAACAATAACTGA
CW666	TGCTTGAAGTGTATATAGGGATCAAA
CW668	CAATAACAACTGGCAAGAAAAGGT

References: **1.** Hiller NL, Janto B, Hogg JS, Boissy R, Yu S, Powell E, Keefe R, Ehrlich NE, Shen K, Hayes J, Barbadora K, Klimke W, Dernovoy D, Tatusova T, Parkhill J, Bentley SD, Post JC, Ehrlich GD, Hu FZ. 2007. Comparative genomic analyses of seventeen *Streptococcus pneumoniae* strains: insights into the pneumococcal supragenome. J Bacteriol 189:8186-95. **2.** Wang CY, Patel N, Wholey WY, Dawid S. 2018. ABC transporter content diversity in *Streptococcus pneumoniae* impacts competence regulation and bacteriocin production. Proc Natl Acad Sci U S A 115:E5776-E5785.
