# Supplementary material for: Molecular Determinants of Substrate Selectivity of a Pneumococcal Rgg-Regulated Peptidase-Containing ABC Transporter
Source: mBio. 2020 Feb 11;11(1):e02502-19. doi: 10.1128/mBio.02502-19 (PMC7018657; doi:10.1128/mBio.02502-19)
Supplement: TEXT S1 [file mBio.02502-19-s0001.pdf]

Supplemental Methods

Chemically Defined Media (CDM+) composition: The following formulation was ordered from Gibco through Thermo-Fisher Scientific using their custom media service:

**Name**	**Concentration (g/L)**
Adenine	0.02
L-alanine	0.1
L-arginine	0.1
L-aspartic acid	0.1
D-biotin	0.0002
Calcium chloride, anhydrous	0.0051
Calcium D-pantothenate	0.002
Cyanocobalamin	0.0001
L-cystine disodium salt	0.0629
Ferric nitrate, nonahydrate	0.001
Ferrous sulfate, heptahydrate	0.005
Folic acid	0.0008
L-glutamic acid	0.1
L-glutamine	0.2
Glycine	0.1
Guanine hydrochloride, anhydrous	0.0182
L-histidine	0.1
trans-4-hydroxy-L-proline	0.1
L-isoleucine	0.1
L-leucine	0.1
L-lysine	0.1
Magnesium sulfate, anhydrous	0.3419
Manganese(II) sulfate, monohydrate	0.005596
L-methionine	0.1
Niacinamide	0.001
Beta-NAD hydrate	0.0025
4-aminobenzoic acid	0.0002
L-phenylalanine	0.1
Potassium phosphate, dibasic, anhydrous	0.2
Potassium phosphate, monobasic, anhydrous	1
L-proline	0.1
Pyridoxal hydrochloride	0.001
Pyridoxine hydrochloride	0.000853
Riboflavin	0.002
L-serine	0.1
Sodium acetate, anhydrous	2.7126
Sodium phosphate, dibasic, anhydrous	7.35
Sodium phosphate, monobasic, monohydrate	3.195
Thiamine hydrochloride	0.001
L-threonine	0.2
L-tryptophan	0.1
L-tyrosine disodium salt	0.144
Uracil	0.02
L-valine	0.1
**Total**	**17.092949**
1.709 g CDM powder, 0.25 g sodium bicarbonate, 0.075 g L-cysteine hydrochloride, and 1 g glucose, 1mL of 10% choline chloride were added to a final volume of 100mL ddH$_2$O and pH adjusted if needed to 7.00. Media was filter sterilized, stored at 4C and used within 3 days.	

DNA manipulation. All PCR reactions for downstream Gibson assembly, transformation, or sequencing applications were performed using Phusion polymerase (NEB, M0530). Other PCR reactions were performed using Taq polymerase (NEB, M0273). For PCR reactions in which cells were used as template, the cells were obtained from starter cultures resuspended in sterile water and added to the PCR reaction at a 1:20 (Phusion) or 1:10 (Taq) dilution. For PCR reactions in which crude Gibson assembly product was used as template, the crude Gibson assembly product was added to the PCR reaction at a 1:100 dilution. Gibson assembly was performed using NEB HiFi DNA Assembly master mix (NEB, E2621). Primers were designed with the aid of primer3 (1, 2) and synthesized by IDT.

Construction of the Janus+ cassette. Two PCR products, amplified using primer pairs CW105/CW357 on Janus cassette (3) template, and CW358/CW106 on Sweet Janus+ cassette (4) template, were ligated via Gibson assembly. The resulting 1402-bp product formed the Janus+ cassette, which contains the *aphA-3* gene (kanamycin resistance) driven by the pneumococcal *amiA* promoter, followed by the *rpsL*+ gene (dominant-negative streptomycin sensitivity) driven by the pneumococcal *rplM* promoter. The version of *rpsL*+ included in Janus+ has the same five silent substitutions as the version in Sweet Janus+. These substitutions and the increased expression from the *rplM* promoter collectively reduce the rate of spontaneous reversion to a streptomycin resistant phenotype in strains carrying the Janus+ cassette compared to the original Janus cassette.

Construction of the Janus2 cassette. A gBlocks DNA fragment was synthesized by IDT containing the gentamicin resistance gene *aacC1* from pPEPY-PF6-*lacI* (5). This template was PCR amplified using primers CW407/CW406 and ligated via Gibson assembly with another PCR product amplified using primers CW105/CW375 from Sweet Janus+ cassette (4) template. The resulting 2069-bp product formed the Janus2 cassette, which contains the *sacB* and *aacC1* genes in a single operon driven by pneumococcal *amiA* promoter.

Allelic exchange using the Janus2 cassette. The Janus2 cassette confers gentamicin resistance (200 µg/mL) and sensitivity to 10% sucrose to pneumococcal strains into which it is inserted. This allows it to be used as a counterselectable marker in the same vein as the Janus (3) and Sweet Janus cassettes (6) on which it is based. Initial selection after transforming the Janus2 cassette into pneumococcus is performed using gentamicin. Afterwards, the cassette can be exchanged with an arbitrary, markerless DNA fragment. Growth in the presence of 10% sucrose selects for transformants that have successfully exchanged the Janus2 cassette. Because the negative selection step for Janus2 does not require a streptomycin resistant copy of *rpsL* in the chromosome (or any other specific feature), Janus2 theoretically can be used in any pneumococcal strain “as is”. Also, the Janus2 cassette and the original Janus cassette (or Janus+ cassette) use orthogonal selection agents. Therefore, Janus2 can be inserted into a strain already harboring Janus, and vice versa. Exchange of the two cassettes can then be performed in either order. Alternatively, insertion and exchange of the cassettes can

each be performed in a single transformation, halving the number of transformation steps required to perform allelic exchange at two unlinked loci.

Construction of the Sp9-BS68 constitutive luciferase reporter. The Sp9-BS68 PF6-*luc* reporter in which the firefly luciferase gene (*luc*) is expressed from the highly active PF6 promoter (5) was created as follows. First, a PCR product (Sp9-BS68-CEP-Janus2-*luc*) of the Janus2 cassette and *luc* inserted into the CEP site (7) between *treR* and *amiF* was amplified using primers CW303/CW295 following Gibson assembly of four PCR products amplified using primer pairs CW456/CW454 on Sp9-BS68 template, CW105/CW406 on Janus2 cassette template, CW457/CW458 on P1666 (4) template, and CW343/CW294 on a GenParts fragment (Genscript) containing the *thrC* terminator from *E. coli* (8), the *tufA* terminator from pneumococcus (9), and approximately 500 bp of flanking sequence including *treR*. The assembled product contains a deletion of the region from n.t. +639 of the gene *CGSSp9BS68_00992* to n.t. −466 of the gene *CGSSp9BS68_00972*, into which are inserted the Janus2 cassette followed immediately by *luc*, oriented in the same direction as *amiF*, then a 50-n.t. spacer and finally the two terminators. The deletion removes an ABC transporter operon predicted to encode a non-functional product due to disruptions of the putative substrate-binding protein and permease genes. Second, a PCR product (Sp9-BS68-CEP-PF6-*luc*) of the luciferase reporter was amplified using primers CW463/CW188 following Gibson assembly of three PCR products amplified using primer pairs CW464/CW455 on Sp9-BS68 template, CW380/CW190 on a GenParts fragment (Genscript) containing the *hisI* and *rpsI* terminators from *E. coli* (8), the PF6 promoter, and the first 103 n.t. of *luc*, and CW191/CW189 on P1666 template. The Sp9-BS68-CEP-Janus2-*luc* PCR product was transformed into Sp9-BS68 to create strain P2769 and then the Janus2 cassette was exchanged with the Sp9-BS68-CEP-PF6-*luc* PCR product to create strain P2772.

Construction of Sp9-BS68 rtg *luciferase reporters.* The Sp9-BS68 P_{rtgA}-*luc* reporter in which the firefly luciferase gene (*luc*) is inserted in place of *rtgA* following an ectopic copy of the *rtgS1*/*rtgA* promoter was created as follows. A PCR product (Sp9-BS68-CEP-P_{rtgA}-*luc*) was amplified using primers CW463/CW188 following Gibson assembly of three PCR products amplified using primer pairs CW464/CW455 on Sp9-BS68 template, CW380/CW190 on a GenParts fragment (Genscript) containing the *hisI* and *rpsI* terminators from *E. coli*, the region from n.t. −429 to n.t. −1 of *rtgA*, and the first 103 n.t. of *luc*, and CW191/CW189 on P1666 template. Then, the Janus2 cassette in P2769 was exchanged with the Sp9-BS68-CEP-P_{rtgA}-*luc* PCR product to create strain P2775. While the ectopic promoter region of *rtgA* in front of *luc* in P2775 contains a copy of *rtgS1*, this copy is disrupted by a frameshift mutation. Therefore, P2775 still only has one functional copy of *rtgS1*.

The Sp9-BS68 P_{rtgS1}-*luc* reporter in which the firefly luciferase gene (*luc*) is inserted in place of *rtgS1* following an ectopic copy of the *rtgS1* promoter was created as follows. A PCR product (Sp9-BS68-CEP-P_{rtgS1}-*luc*) was amplified using primers CW463/CW188 following Gibson assembly of two PCR products amplified using primer pairs CW464/CW494 and CW158/CW189 on P2775 template. Then, the Janus2 cassette in P2769 was exchanged with the Sp9-BS68-CEP-P_{rtgS1}-*luc* PCR product to create strain P2792.

Construction of D39 rtg *luciferase reporter.* The D39 P_{rtgS1}-*luc* reporter in which the firefly luciferase gene (*luc*) is inserted in place of *rtgS1* following an ectopic copy of the *rtgS1* promoter was created as follows. First, a PCR product (D39-CEP-Janus2-*luc*) of the Janus2 cassette and *luc* inserted into the CEP site between *treR* and *amiF* was amplified using primers CW463/CW293 following Gibson assembly of three PCR products amplified using primer pairs CW464/CW471 on P2055 (4) template, CW105/CW474 on P2769 template, and CW481/CW292 on P2055 template. The assembled product contains a deletion of the region from n.t. −101 to n.t. +1168 of the gene *SPD_1666*, replaced by the insertion from P2769. The deletion removes a degenerate transposon. Second, a PCR product (Sp9-BS68-CEP-P_{rtgS1}-*luc*) containing the luciferase reporter was amplified using primers CW463/CW188 following Gibson assembly of three PCR products amplified using primer pairs CW464/CW482 on P2055 template, CW380/CW494 on P2775 template, and CW158/CW189 on P1666 template. The D39-CEP-Janus2-*luc* PCR product was transformed into P2055 to create strain P2779 and then the Janus2 cassette was exchanged with the D39-CEP-P_{rtgS1}-*luc* PCR product to create strain P2790.

Construction of Sp9-BS68 rtgR *and* rtgS1 *deletion mutants.* The Sp9-BS68 Δ*rtgR* strain was created as follows. First, a PCR product (Sp9-BS68-*rtgR*-Janus2) was amplified using primers CW487/CW486 following Gibson assembly of three PCR products amplified using primer pairs CW488/CW490 on Sp9-BS68 template, CW105/CW406 on Janus2 cassette template, and CW489/CW485 on Sp9-BS68 template. The assembled product contains the Janus2 cassette inserted in place of *rtgR*. Second, a PCR product (Sp9-BS68-Δ*rtgR*) was amplified using primers CW487/CW486 following Gibson assembly of two PCR products amplified using primer pairs CW488/CW491 and CW492/CW485 on Sp9-BS68 template. The Sp9-BS68-*rtgR*-Janus2 PCR product was transformed into P2792 and then the Janus2 cassette was exchanged with the Sp9-BS68-Δ*rtgR* PCR product to create strain P2802.

The Sp9-BS68 Δ*rtgS1* strain was created as follows. First, a PCR product (Sp9-BS68-*rtgR*-*rtgS1*-Janus2) was amplified using primers CW498/CW486 following Gibson assembly of three PCR products amplified using primer pairs CW499/CW523 on Sp9-BS68 template, CW105/CW406 on Janus2 cassette template, and CW489/CW485 on Sp9-BS68 template. The assembled product contains the Janus2 cassette inserted in place of *rtgR* and *rtgS1*. Second, a PCR product (Sp9-BS68-Δ*rtgS1*) was amplified using primers CW498/CW486 following Gibson assembly of three PCR products amplified using primer pairs CW488/CW503 on Sp9-BS68 template, CW502/CW484 on a gBlocks fragment (IDT) containing an in-frame deletion of n.t. +31 to n.t. +84 of *rtgS1*, and CW483/CW485 on Sp9-BS68 template. The Sp9-BS68-*rtgR*-*rtgS1*-Janus2 PCR product was transformed into P2792 to create strain P2798 and then the Janus2 cassette was exchanged with the Sp9-BS68-Δ*rtgS1* PCR product to create strain P2804.

The Sp9-BS68 Δ*rtgR*Δ*rtgS1* strain was created as follows. A PCR product (Sp9-BS68-Δ*rtgR*Δ*rtgS1*) was amplified using primers CW498/CW486 following Gibson assembly of two PCR products amplified using primer pairs CW488/CW484 on P2804

template and CW483/CW485 on P2802 template. Then, the Janus2 cassette in P2798 was exchanged with the Sp9-BS68-Δ*rtgR*Δ*rtgS1* PCR product to create strain P2811.

Construction of D39 rtgS1 *and* rtgS2 *deletion mutants.* The D39 Δ*rtgS1* strain was created as follows. First, a PCR product (D39-*rtgS1*-Janus2) was amplified using primers CW556/CW498 following Gibson assembly of three PCR products amplified using primer pairs CW496/CW500 on P2055 template, CW105/CW406 on Janus2 cassette template, and CW501/CW499 on P2055 template. Second, a PCR product (D39-Δ*rtgS1*) was amplified using primers CW556/CW498 following Gibson assembly of three PCR products amplified using primer pairs CW496/CW483 on P2055 template, CW484/CW502 on P2804 template, and CW503/CW499 on P2055 template. The D39-*rtgS1*-Janus2 PCR product was transformed into P2790 to create strain P2851 and then the Janus2 cassette was exchanged with the D39-Δ*rtgS1* PCR product to create strain P2859.

The D39 Δ*rtgS2* strain was created as follows. First, a PCR product (D39-*rtgS2*-Janus+) was amplified using primers CW513/CW518 following Gibson assembly of three PCR products amplified using primer pairs CW548/CW562 on P2055 template, CW105/CW106 on Janus+ cassette template, and CW561/CW519 on P2055 template. Second, a PCR product (D39-Δ*rtgS2*) was amplified using primers CW513/CW518 following Gibson assembly of three PCR products amplified using primer pairs CW548/CW483 on P2055 template, CW484/CW520 on a gBlocks fragment (IDT) containing an in-frame deletion of n.t. +31 to n.t. +84 of *rtgS2*, and CW521/CW519 on P2055 template. Third, a PCR product (D39-*rtgS1*) was amplified using primers CW556/CW498 on P2055 template. The D39-*rtgS2*-Janus+ PCR product was transformed into P2851 to create strain P2896. Then, the Janus+ cassette was exchanged with the D39-Δ*rtgS2* PCR product to create strain P2902. Finally, the Janus2 cassette was exchanged with the D39-*rtgS1* PCR product to create strain P2908.

The D39 Δ*rtgS1*Δ*rtgS2* strain was created as follows. The Janus2 cassette in strain P2902 was exchanged with the D39-Δ*rtgS1* PCR product to create strain P2910.

Construction of Sp9-BS68 ami *and* ppt *deletion mutants.* The Sp9-BS68 Δ*amiCD* strain was created as follows. First, a PCR product (Sp9-BS68-*amiCD*-Janus2) was amplified using primers CW616/CW607 following Gibson assembly of three PCR products amplified using primer pairs CW617/CW614 on Sp9-BS68 template, CW105/CW406 on Janus2 cassette template, and CW611/CW606 on Sp9-BS68 template. Second, a PCR product (Sp9-BS68-Δ*amiCD*) was amplified using primers CW616/CW607 following Gibson assembly of two PCR products amplified using primer pairs CW617/CW613 and CW612/CW606 on Sp9-BS68 template. The Sp9-BS68-*amiCD*-Janus2 PCR product was transformed into P2792, and then the Janus2 cassette was exchanged with the Sp9-BS68-Δ*amiCD* PCR product to create strain P3075.

The Sp9-BS68 Δ*pptAB* strain was created as follows. First, a PCR product (Sp9-BS68-*pptAB*-Janus2) was amplified using primers CW619/CW627 following Gibson assembly of three PCR products amplified using primer pairs CW618/CW622 on Sp9-BS68 template, CW105/CW406 on Janus2 cassette template, and CW625/CW628 on Sp9-BS68 template. Second, a PCR product (Sp9-BS68-Δ*amiCD*) was amplified using

primers CW619/CW627 following Gibson assembly of two PCR products amplified using primer pairs CW618/CW623 and CW624/CW628 on Sp9-BS68 template. The Sp9-BS68-*pptAB*-Janus2 PCR product was transformed into P2792, and then the Janus2 cassette was exchanged with the Sp9-BS68-Δ*pptAB* PCR product to create strain P3077.

Construction of Sp9-BS68 rtgS1 *promoter mutation reporters.* The Sp9-BS68 P_{rtgS1}(P2)-*luc* reporter strain was created as follows. First, the Sp9-BS68-CEP-Janus2-*luc* PCR product was amplified using primers CW463/CW468 on P2769 template. Second, a PCR product (Sp9-BS68-CEP-P_{rtgS1}(P2)-*luc*) was amplified using primers CW463/CW188 following Gibson assembly of two PCR products amplified using primer pairs CW464/CW570 and CW629/CW189 on P2792 template. The Sp9-BS68-CEP-Janus2-*luc* PCR product was transformed into strain P2804 to create strain P3080, and then the Janus2 cassette was exchanged with the Sp9-BS68-CEP-P_{rtgS1}(P2)-*luc* PCR product to create strain P3100.

The Sp9-BS68 P_{rtgS1}(P2)-*luc* reporter strains with promoter sequence mutations were created as follows. The mut1, mut2, mut3, and mut4 PCR products were amplified using primers CW463/CW188 following Gibson assembly of the following pairs of PCR products, all amplified from P3100 template: mut1, CW464/CW632 and CW633/CW189; mut2, CW464/CW630 and CW631/CW189; mut3, CW464/CW634 and CW635/CW189; mut4, CW464/CW636 and CW637/CW189. The Janus2 cassette in strain P3080 was then exchanged with mut1, mut2, mut3, and mut4 to create strains P3123, P3125, P3127, and P3129, respectively.

Construction of D39 com/blp *luciferase reporters with repaired* rtgA *(RtgAB$^+$).* First, a PCR product (D39-*rtgAXB*-SJanus+) was amplified using primers CW556/CW557 following Gibson assembly of three PCR products amplified using primer pairs CW496/CW537 on D39 template, CW105/CW106 on Sweet Janus+ cassette template, and CW509/CW559 on D39 template. Second, a PCR product (D39-$rtgAXB_{Sp9\text{-}BS68}$) was amplified using primers CW556/CW557 following Gibson assembly of three PCR products amplified using primer pairs CW496/CW502 on D39 template, CW503/CW511 on Sp9-BS68 template, and CW522/CW559 on D39 template. The D39-*rtgAXB*-SJanus+ PCR product was transformed into strains P2665, P2666, P2668, and P2670 and then the Sweet Janus+ cassettes were exchanged with the D39-$rtgAXB_{Sp9\text{-}BS68}$ PCR product to create strains P2838, P2840, P2842, and P2844, respectively.

Construction of Sp9-BS68 strains for mouse colonization assays. The spectinomycin-resistant Sp9-BS68 strains were created as follows. A PCR product (Sp9-BS68-CEP-spcR) was amplified using primers CW463/CW468 following Gibson assembly of three PCR products amplified using primer pairs CW464/CW570 on P2792 template, CW571/CW572 on pE81 template, and CW343/CW292 on P2792 template. The Sp9-BS68-CEP-spcR PCR product was transformed into strains P2792 and P2811 to create strains P3001 and P3003, respectively.

The gentamicin-resistant Sp9-BS68 strain was created as follows. A PCR product (Sp9-BS68-CEP-genR) was amplified using primers CW463/CW468 following Gibson assembly of three PCR products amplified using primer pairs CW464/CW600 on

P3001 template, CW598/CW599 on Janus2 cassette template, and CW343/CW292 on P3001 template. The Sp9-BS68-CEP-genR PCR product was transformed into strain P2811 to create strain P3025.

Mouse-passaged versions of the above strains were created as follows. P3001, P3003, and P3025 were inoculated into 5-7-week-old female BALB/c mice using the same protocol as that used for the single-strain colonization assays. After 24 hours, the colonizers were recovered via nasal wash using the same protocol as that used for the colonization assays. Nasal washes were plated on spectinomycin- or gentamicin-containing TSA plates supplemented with 5 µg/mL catalase and incubated overnight at 37°C with 5% CO_2. From each of the P3001, P3003, and P3025-inoculated mice, eight individual colonies were pooled to create stocks of strains P3035, P3037, and P3039, respectively. These mouse-passaged strains were then used for the mouse colonization assays.

Construction of rtg *autoinducing-deficient tagged BlpI expressing R6 strains.* First, a PCR product (R6-*rtgS1*-*rtgD2*-SJanus+) was amplified using primers CW556/CW550 following Gibson assembly of three PCR products amplified using primer pairs CW496/CW500 on R6 template, CW105/CW106 on Sweet Janus+ cassette template, and CW538/CW551 on R6 template. Second, a PCR product (R6-Δ*rtgS1*-*rtgAXB*) was amplified using primers CW556/CW550 following Gibson assembly of two PCR products amplified using primers CW556/CW565 on P2859 template, and CW542/CW551 on R6 template. Third, a PCR product (R6-Δ*rtgS1*-$rtgAXB_{Sp9\text{-}BS68}$) was amplified using primers CW556/CW550 following Gibson assembly of three PCR products amplified using primers CW556/CW483 on R6 template, CW484/CW541 on P2804 template, and CW542/CW551 on R6 template. The R6-*rtgS1*-*rtgD2*-SJanus+ PCR product was transformed into strains P2538, P2565, P2567, and P2569 to create strains P2865, P2867, P2869, and P2871, respectively. Then, the Sweet Janus+ cassettes in P2865, P2867, P2869, and P2871 were exchanged for the R6-Δ*rtgS1*-*rtgAXB* PCR product to create strains P2878, P2880, P2884, and P2888, respectively. Finally, the Sweet Janus+ cassette in strain P2867 was exchanged for the R6-Δ*rtgS1*-$rtgAXB_{Sp9\text{-}BS68}$ PCR product to create strain P2882.

Construction of RtgC-HiBiT and RtgG-HiBiT expressing R6 strains. The R6 strains expressing RtgC-HiBiT were created as follows. First, the pE57 insertion and $blpI_{P133}$-*HiBiT* genes were removed from P2880, P2882, P2884, and P2888. A PCR product (P654-*blpA*-*pncS*-Janus+) was amplified using primers CW271/CW219 following Gibson assembly of three PCR products amplified using primer pairs CW272/CW270 on P654 (10) template, CW105/CW106 on Janus+ cassette template, and CW238/CW234 on P654 template. Another PCR product (P654-ΔpE57) was amplified using primers CW585/CW219 following Gibson assembly of two PCR products amplified using primer pairs CW584/CW586 and CW587/CW234 on P654 template. The P654-*blpA*-*pncS*-Janus+ PCR product was transformed into strains P2880, P2882, P2884, and P2888 and then exchanged with the P654-ΔpE57 PCR product to create strains P2934, P2936, P2938, and P2940, respectively. Second, *rtgC-HiBiT* was inserted downstream of *rtgB* in these strains. A PCR product (P2880-*rtgC*-*rtgD2*-SJanus+) was amplified using primers CW451/CW550 following Gibson assembly of three PCR products

amplified using primer pairs CW531/CW534 on R6 template, CW105/CW106 on Sweet Janus+ cassette template, and CW538/CW551 on R6 template. Another PCR product (P2882-*rtgC*-*rtgD2*-SJanus+) was amplified using primers CW451/CW550 following Gibson assembly of three PCR products amplified using primer pairs CW543/CW546 on Sp9-BS68 template, CW105/CW106 on Sweet Janus+ cassette template, and CW538/CW551 on R6 template. A third PCR product (P2880-*rtgC-HiBiT*) was amplified using primers CW451/CW550 following Gibson assembly of three PCR products amplified using primer pairs CW531/CW506 on R6 template, CW592/CW593 on a gBlocks fragment (IDT) containing $rtgC_{R6}$ with an insertion of sequence encoding HiBiT tag preceded by a 10-residue linker (ggtggtggaggttcaggaggtggaggttctgtttctggttggcgtctttttaaaaaaatttca) followed by $rtgD1_{R6}$, and CW594/CW551 on R6 template. A final PCR product (P2882-*rtgC-HiBiT*) was amplified using primers CW451/CW550 following Gibson assembly of three PCR products amplified using primer pairs CW543/CW546 on a template containing *rtgAXB* from Sp9-BS68 inserted in place of *rtgAXB* in the D39 *rtg* locus, CW592/CW593 on the *rtgC-HiBiT* gBlocks fragment from above, and CW594/CW551 on R6 template. The P2880-*rtgC*-*rtgD2*-SJanus+ PCR product was transformed into strains P2934, P2938, and P2940 to create strains P2945, P2949, and P2951, respectively. The P2882-*rtgC*-*rtgD2*-SJanus+ PCR product was transformed into strain P2936 to create strain P2947. Then, the Sweet Janus+ cassettes in strains P2945, P2949, and P2951 were exchanged for the P2880-*rtgC-HiBiT* PCR product to create strains P2959, P2963, and P2965, respectively. Finally, the Sweet Janus+ cassette in strain P2947 was exchanged for the P2882-*rtgC-HiBiT* PCR product to create strain P2961.

The R6 strains expressing RtgG-HiBiT were created as follows. A PCR product (P2880-*rtgG-HiBiT*) was amplified using primers CW451/CW550 following Gibson assembly of three PCR products amplified using primer pairs CW531/CW595 on R6 template, CW522/CW593 on a gBlocks fragment (IDT) containing $rtgG_{Sp9\text{-}BS68}$ with an insertion of sequence encoding HiBiT tag preceded by a 10-residue linker (ggtggtggaggttcaggaggtggaggttctgtttctggttggcgtctttttaaaaaaatttca) followed by $rtgH_{Sp9\text{-}BS68}$, and CW594/CW551 on R6 template. Another PCR product (P2882-*rtgG-HiBiT*) was amplified using primers CW451/CW550 following Gibson assembly of three PCR products amplified using primer pairs CW543/CW511 on P2882 template, CW522/CW593 on the *rtgG-HiBiT* gBlocks fragment from above, and CW594/CW551 on R6 template. The Sweet Janus+ cassettes in strains P2945, P2949, and P2951 were exchanged for the P2880-*rtgG-HiBiT* PCR product to create strains P2967, P2971, and P2973, respectively. The Sweet Janus+ cassette in strain P2947 was exchanged for the P2882-*rtgG-HiBiT* PCR product to create strain P2969.

Construction of the R6 strain with $rtgA_{ATG>ATT}$ *expressing RtgG-HiBiT.* A PCR product (P2882-*rtgA*-Janus2) was amplified using primers CW556/CW659 following Gibson assembly of three PCR products amplified using primer pairs CW496/CW537 on P2882 template, CW105/CW406 on Janus2 cassette template, and CW657/CW660 on P2882 template. Another PCR product (P2882-$rtgA_{ATG>ATT}$) was amplified using primers CW556/CW659 following Gibson assembly of three PCR products amplified using primer pairs CW496/CW502 on P2882 template, CW503/CW446 on R6 template, and CW447/CW660 on P2882 template. The P2882-*rtgA*-Janus2 PCR product was

transformed into P2969, and then the Janus2 cassette was exchanged with the P2882-*rtgA*$_{ATG>ATT}$ PCR product to create strain P3162.

Construction of R6 strains expressing BlpI-HiBiT from downstream of rtgB. A PCR product (P2880-*blpI-HiBiT*) was amplified using primers CW451/CW550 following Gibson assembly of three PCR products amplified using primer pairs CW531/CW595 on R6 template, CW522/CW596 on a gBlocks fragment (IDT) containing *blpI-HiBiT*, and CW597/CW551 on P2967 template. Another PCR product (P2882-*blpI-HiBiT*) was amplified using primers CW451/CW550 following Gibson assembly of three PCR products amplified using primer pairs CW543/CW511 on P2882 template, CW522/CW596 on the *blpI-HiBiT* gBlocks fragment from above, and CW597/CW551 on P2967 template. The Sweet Janus+ cassettes in strains P2945, P2949, and P2951 were exchanged for the P2880-*blpI-HiBiT* PCR product to create strains P3027, P3031, and P3033, respectively. The Sweet Janus+ cassette in strain P2947 was exchanged for the P2882-*blpI-HiBiT* PCR product to create strain P3029.

Construction of R6 strains expressing signal sequence-swapped RtgG-HiBiT and BlpI-HiBiT. The R6 strains expressing SS_{RtgG}-BlpI-HiBiT were created as follows. A PCR product (P2880-*SS*$_{rtgG}$*-blpI-HiBiT*) was amplified using primers CW451/CW550 following Gibson assembly of three PCR products amplified using primer pairs CW531/CW595 on R6 template, CW522/CW596 on a gBlocks fragment (IDT) containing *SS*$_{rtgG}$*-blpI-HiBiT*, and CW597/CW551 on P2967 template. Another PCR product (P2882-*SS*$_{rtgG}$*-blpI-HiBiT*) was amplified using primers CW451/CW550 following Gibson assembly of three PCR products amplified using primer pairs CW543/CW511 on P2882 template, CW522/CW596 on the *SS*$_{rtgG}$*-blpI-HiBiT* gBlocks fragment from above, and CW597/CW551 on P2967 template. The Sweet Janus+ cassettes in strains P2945, P2949, and P2951 were exchanged for the P2880-*SS*$_{rtgG}$*-blpI-HiBiT* PCR product to create strains P2981, P2985, and P2987, respectively. The Sweet Janus+ cassette in strain P2947 was exchanged for the P2882-*SS*$_{rtgG}$*-blpI-HiBiT* PCR product to create strain P2983.

The R6 strains expressing SS_{BlpI}-RtgG-HiBiT were created as follows. A PCR product (P2880-*SS*$_{blpI}$*-rtgG-HiBiT*) was amplified using primers CW451/CW550 following Gibson assembly of three PCR products amplified using primer pairs CW531/CW595 on R6 template, CW522/CW596 on a gBlocks fragment (IDT) containing *SS*$_{blpI}$*-rtgG-HiBiT*, and CW597/CW551 on P2967 template. Another PCR product (P2882-*SS*$_{blpI}$*-rtgG-HiBiT*) was amplified using primers CW451/CW550 following Gibson assembly of three PCR products amplified using primer pairs CW543/CW511 on P2882 template, CW522/CW596 on the *SS*$_{blpI}$*-rtgG-HiBiT* gBlocks fragment from above, and CW597/CW551 on P2967 template. The Sweet Janus+ cassettes in strains P2945, P2949, and P2951 were exchanged for the P2880-*SS*$_{blpI}$*-rtgG-HiBiT* PCR product to create strains P2993, P2997, and P2999, respectively. The Sweet Janus+ cassette in strain P2947 was exchanged for the P2882-*SS*$_{blpI}$*-rtgG-HiBiT* PCR product to create strain P2995.

Construction of R6 strains expressing HiBiT-tagged BlpI cargo peptide fused to various signal sequences. The R6 strains expressing SS_{RtgG}(F/M/L/V)-BlpI-HiBiT were created

as follows. A PCR product (P2880-SS_{rtgG}*(F/M/L/V)-blpI-HiBiT*) was amplified using primers CW451/CW550 following Gibson assembly of three PCR products amplified using primer pairs CW531/CW595 on R6 template, CW522/CW596 on a gBlocks fragment (IDT) containing SS_{rtgG}*(F/M/L/V)-blpI-HiBiT*, and CW597/CW551 on P2967 template. Another PCR product (P2882-SS_{rtgG}*(F/M/L/V)-blpI-HiBiT*) was amplified using primers CW451/CW550 following Gibson assembly of three PCR products amplified using primer pairs CW543/CW511 on P2882 template, CW522/CW596 on the SS_{rtgG}*(F/M/L/V)-blpI-HiBiT* gBlocks fragment from above, and CW597/CW551 on P2967 template. The Sweet Janus+ cassettes in strains P2945, P2949, and P2951 were exchanged for the P2880-SS_{rtgG}*(F/M/L/V)-blpI-HiBiT* PCR product to create strains P3009, P3013, and P3015, respectively. The Sweet Janus+ cassette in strain P2947 was exchanged for the P2882-SS_{rtgG}*(F/M/L/V)-blpI-HiBiT* PCR product to create strain P3011.

The R6 strains expressing SS_{BlpI}(Y/L/M/L)-BlpI-HiBiT were created as follows. A PCR product (P2880-SS_{blpI}*(Y/L/M/L)-blpI-HiBiT*) was amplified using primers CW451/CW550 following Gibson assembly of three PCR products amplified using primer pairs CW531/CW595 on R6 template, CW522/CW596 on a gBlocks fragment (IDT) containing SS_{blpI}*(Y/L/M/L)-blpI-HiBiT*, and CW597/CW551 on P2967 template. Another PCR product (P2882-SS_{blpI}*(Y/L/M/L)-blpI-HiBiT* was amplified using primers CW451/CW550 following Gibson assembly of three PCR products amplified using primer pairs CW543/CW511 on P2882 template, CW522/CW596 on the SS_{blpI}*(Y/L/M/L)-blpI-HiBiT* gBlocks fragment from above, and CW597/CW551 on P2967 template. The Sweet Janus+ cassettes in strains P2945, P2949, and P2951 were exchanged for the P2880-SS_{blpI}*(Y/L/M/L)-blpI-HiBiT* PCR product to create strains P3017, P3021, and P3023, respectively. The Sweet Janus+ cassette in strain P2947 was exchanged for the P2882-SS_{blpI}*(Y/L/M/L)-blpI-HiBiT* PCR product to create strain P3019.

The R6 strains expressing SS_{RtgG}(A/A/A/A)-BlpI-HiBiT were created as follows. A PCR product (P2880-SS_{rtgG}*(A/A/A/A)-blpI-HiBiT*) was amplified using primers CW451/CW550 following Gibson assembly of three PCR products amplified using primer pairs CW531/CW595 on R6 template, CW522/CW596 on a gBlocks fragment (IDT) containing SS_{rtgG}*(A/A/A/A)-blpI-HiBiT*, and CW597/CW551 on P2967 template. Another PCR product (P2882-SS_{rtgG}*(A/A/A/A)-blpI-HiBiT*) was amplified using primers CW451/CW550 following Gibson assembly of three PCR products amplified using primer pairs CW543/CW511 on P2882 template, CW522/CW596 on the SS_{rtgG}*(A/A/A/A)-blpI-HiBiT* gBlocks fragment from above, and CW597/CW551 on P2967 template. The Sweet Janus+ cassettes in strain P2945 was exchanged for the P2880-SS_{rtgG}*(A/A/A/A)-blpI-HiBiT* PCR product to create strain P3111. The Sweet Janus+ cassette in strain P2947 was exchanged for the P2882-SS_{rtgG}*(A/A/A/A)-blpI-HiBiT* PCR product to create strain P3113.

The R6 strains expressing SS_{BlpI}(A/A/A/A)-BlpI-HiBiT were created as follows. A PCR product (P2880-SS_{blpI}*(A/A/A/A)-blpI-HiBiT*) was amplified using primers CW451/CW550 following Gibson assembly of three PCR products amplified using primer pairs CW531/CW595 on R6 template, CW522/CW596 on a gBlocks fragment (IDT) containing SS_{blpI}*(A/A/A/A)-blpI-HiBiT*, and CW597/CW551 on P2967 template. Then, the Sweet Janus+ cassettes in strains P2945, P2949, and P2951 were

exchanged for the P2880-*SS$_{blpI}$(A/A/A/A)-blpI-HiBiT* PCR product to create strains P3142, P3144, and P3146, respectively.

The R6 strains expressing SS$_{RtgG}$(N6$_{BlpI}$)-BlpI-HiBiT were created as follows. A PCR product (P2880-*SS$_{rtgG}$(N6$_{blpI}$)-blpI-HiBiT*) was amplified using primers CW451/CW550 following Gibson assembly of three PCR products amplified using primer pairs CW531/CW595 on R6 template, CW522/CW596 on a gBlocks fragment (IDT) containing *SS$_{rtgG}$(N6$_{blpI}$)-blpI-HiBiT*, and CW597/CW551 on P2967 template. Another PCR product (P2882-*SS$_{rtgG}$(N6$_{blpI}$)-blpI-HiBiT*) was amplified using primers CW451/CW550 following Gibson assembly of three PCR products amplified using primer pairs CW543/CW511 on P2882 template, CW522/CW596 on the *SS$_{rtgG}$(N6$_{blpI}$)-blpI-HiBiT* gBlocks fragment from above, and CW597/CW551 on P2967 template. The Sweet Janus+ cassettes in strains P2945, P2949, and P2951 were exchanged for the P2880-*SS$_{rtgG}$(N6$_{blpI}$)-blpI-HiBiT* PCR product to create strains P3055, P3059, and P3061, respectively. The Sweet Janus+ cassette in strain P2947 was exchanged for the P2882-*SS$_{rtgG}$(N6$_{blpI}$)-blpI-HiBiT* PCR product to create strain P3057.

The R6 strains expressing SS$_{BlpI}$(N6$_{RtgG}$)-BlpI-HiBiT were created as follows. A PCR product (P2880-*SS$_{blpI}$(N6$_{rtgG}$)-blpI-HiBiT*) was amplified using primers CW451/CW550 following Gibson assembly of three PCR products amplified using primer pairs CW531/CW595 on R6 template, CW522/CW596 on a gBlocks fragment (IDT) containing *SS$_{blpI}$(N6$_{rtgG}$)-blpI-HiBiT*, and CW597/CW551 on P2967 template. Another PCR product (P2882-*SS$_{blpI}$(N6$_{rtgG}$)-blpI-HiBiT*) was amplified using primers CW451/CW550 following Gibson assembly of three PCR products amplified using primer pairs CW543/CW511 on P2882 template, CW522/CW596 on the *SS$_{blpI}$(N6$_{rtgG}$)-blpI-HiBiT* gBlocks fragment from above, and CW597/CW551 on P2967 template. The Sweet Janus+ cassettes in strains P2945, P2949, and P2951 were exchanged for the P2880-*SS$_{blpI}$(N6$_{rtgG}$)-blpI-HiBiT* PCR product to create strains P3081, P3085, and P3087, respectively. The Sweet Janus+ cassette in strain P2947 was exchanged for the P2882-*SS$_{blpI}$(N6$_{rtgG}$)-blpI-HiBiT* PCR product to create strain P3083.

The R6 strains expressing SS$_{RtgG}$[ΔN(2-6)]-BlpI-HiBiT or SS$_{BlpI-ΔN(2-6)}$-BlpI-HiBiT were created as follows. A PCR product (P2882-*SS$_{rtgG}$[ΔN(2-6)]-blpI-HiBiT*) was amplified using primers CW451/CW550 following Gibson assembly of three PCR products amplified using primer pairs CW543/CW595 on P2882 template, CW522/CW596 on a gBlocks fragment (IDT) containing *SS$_{rtgG}$[ΔN(2-6)]-blpI-HiBiT*, and CW597/CW551 on P2967 template. Another PCR product (P2882-*SS$_{blpI}$[ΔN(2-6)]-blpI-HiBiT*) was amplified using primers CW451/CW550 following Gibson assembly of three PCR products amplified using primer pairs CW543/CW511 on P2882 template, CW522/CW596 on a gBlocks fragment (IDT) containing *SS$_{blpI}$[ΔN(2-6)]-blpI-HiBiT*, and CW597/CW551 on P2967 template. The Sweet Janus+ cassette in strain P2947 was exchanged for the P2882-*SS$_{rtgG}$[ΔN(2-6)]-blpI-HiBiT* and P2882-*SS$_{blpI}$[ΔN(2-6)]-blpI-HiBiT* PCR products to create strains P3166 and P3170, respectively.

The R6 strains expressing SS$_{RtgG}$-BlpI-HiBiT with single-residue substitutions in the signal sequence were created as follows. PCR products were amplified using primers CW451/CW550, each following Gibson assembly of two PCR products amplified using the following primer pairs on P2983 template: CW543/CW655 and CW656/CW551 [E(−22)A], CW543/CW654 and CW656/CW551 [L(−21)A], CW543/CW653 and CW656/CW551 [I(−20)A], CW543/CW652 and CW656/CW551

[L(−19)A], and CW543/CW649 and CW651/CW551 [P(−18)M]. The Sweet Janus+ cassette in strain P2947 was exchanged for the above PCR products to create strains P3133, P3135, P3137, P3138, and P3160, respectively.

Sequencing of the Sp9-BS68 rtg *locus.* To bridge gaps in the *rtg* locus in the published genome sequence of Sp9-BS68 (11), we amplified the region between *rtgG* and *rtgD2* using primers CW545/CW539 and performed Sanger sequencing using primers CW640, CW665, CW518, CW666, CW668, CW568, and CW593. This allowed us to manually and unambiguously join contigs 306 (ABAB01000022), 342 (ABAB01000053), 99 (ABAB01000059), and 173 (ABAB01000005).

P_{rtgA}-luc *lytic luciferase reporter assay.* Cells expressing P_{rtgA}-*luc* reporters were grown in THY, CDM+, or RPMI (Thermo Fisher Scientific, 11875093) ± 1% fetal bovine serum (Thermo Fisher Scientific, 10437028). At OD_{620} 0.2, cells were harvested, pelleted by centrifugation at 6000×*g*, 5 min, 4°C, washed with PBS, pelleted again and resuspended in lysis buffer (50 mM Tris-HCl pH 7.5, 10 mM $MgCl_2$, 1 mM DTT, 0.2% Triton X-100). After incubation with lysis buffer for 15 min at room temperature, the lysed samples were mixed with firefly luciferase reaction buffer [50 mM Tris-HCl pH 7.5, 10 mM $MgCl_2$, 1 mM DTT, 2 mM firefly luciferin (Thermo Fisher Scientific, 88294), 2 mM ATP, 1 mM coenzyme A] at a 1:1 ratio and luminescence was immediately read with a Synergy HTX plate reader (Biotek) in a white 96-well plate (Costar, 3917). Differences between groups were assessed by ANOVA followed by Tukey's HSD test, using the aov() and TukeyHSD() functions in R 3.5.1.

Calculations for rtg*,* com*, and* blp *activation events.* Adapted from ref (4). Analysis was restricted to timepoints at which OD_{620} was greater than or equal to 0.01. The low values of both luminescence and OD_{620} at timepoints before this cell density threshold resulted in low signal-to-noise ratios that made automated analysis difficult. An activation event was defined as the first timepoint at which the activation level remained above a threshold T for at least three consecutive readings. T was defined as a function of cell density as follows:

$$T = \frac{T^0}{\sqrt{N}}$$

T^0, threshold constant; N, cell density (OD_{620}). This function was chosen because it was the simplest form that empirically yielded a threshold curve with high sensitivity and specificity. The constant T^0 was empirically determined by manual inspection of activation level curves from pheromone-treated samples as positive controls and curves from non-treated pheromone deletion strains as negative controls. Wells that were already activated at the beginning of the analysis period were left censored and wells that did not activate before they reached their maximum observed cell densities were right censored.

Peptide secretion assays. Samples for extracellular peptide quantification were read with a Synergy HTX plate reader (Biotek) in a white 96-well plate (Costar, 3917) following addition of HiBiT Extracellular Detection Reagent (Promega, N2421) at a 1:1 ratio. For endpoint assays, each sample was quantified with three technical replicates

and read 5 min following reagent addition. For time-course assays, each sample was quantified with one technical replicate and read 1 min following reagent addition. Samples for intracellular peptide quantification were pelleted at 6000×*g*, 5 min, 4°C and resuspended in proteinase K buffer [20 mM MES pH 6.5, 20 mM $MgCl_2$, 0.5 M sucrose, 100 µg/mL proteinase K (Fisher Scientific, BP1700)]. A 15-min incubation at 37°C removed residual extracellular peptide by proteinase K digestion. Afterwards, proteinase K was inactivated by addition of 1 mM phenylmethanesulfonyl fluoride (Calbiochem, 7110). Cells were lysed by addition of 1% Triton X-100 followed by incubation at room temperature for 15 min. The lysed samples were then mixed with HiBiT Extracellular Detection Reagent at a 1:1 ratio in a white 96-well plate and read with a Synergy HTX plate reader. Each sample was quantified with three technical replicates and read 5 min following reagent addition.

Genomic analysis of rtg*.* Genomes of the Massachusetts pneumococcal isolate collection (BioProject Accession: PRJEB2632) were filtered based on sequence coverage of the predicted location of *rtg*. Three possible upstream flanking genes were defined: a gene coding for a LysM-domain protein (*SPD_0104*), *argG* (*SPD_0110*), and *argH* (*SPD_0111*). Two possible downstream flanking genes were defined: a putative endoRNAse gene (*SPD_0125*; disrupted in D39) and *pspA* (*SPD_0126*). The D39 versions of these genes were BLASTed (megablast profile) against all genomes in the Massachusetts collection. In the case of *pspA*, only the first 250 nucleotides were used so that the choline-binding repeats found later in the gene did not complicate the analysis. All genomes in which at least one upstream flanking gene was found on the same contig as at least one downstream flanking gene were chosen for further analysis (318 in total); all other genomes were discarded. For each genome in the filtered set of 318, the sequence between the two closest flanking genes was extracted and a distance matrix for these sequences representing *rtg* loci was calculated using BIGSdb (12). The set of unique *rtg* genes found in Sp9-BS68 and D39 was used as the reference gene list. Then, a neighbor-joining tree was constructed from the distance matrix using SplitsTree (13). This tree was used to guide manual clustering of the *rtg* loci into different groups based on gene presence and synteny. New unique *rtg* genes found in the course of this analysis were added to the initial set of genes from Sp9-BS68 and D39 and the updated set was BLASTed (blastn profile) against all 616 genomes in the Massachusetts collection to determine how many strains in the full collection encoded *rtg*. Genomes with a hit (≥ 80% query coverage, ≥ 70% sequence identity) for at least one *rtg* gene were considered to be *rtg*-positive.

Calculations for mouse colonization assays. The competitive nasal colonization performed for this publication was part of an approved protocol authorized by the University of Michigan IACUC. Ten-fold serial dilutions of PBS suspensions used to inoculate mice were spot plated (5 µL per spot, 3 replicates per sample) on tryptic soy agar (TSA) plates supplemented with 5 µg/mL catalase and containing either neomycin (selects for all pneumococcus), spectinomycin, or gentamicin. Plates were incubated at 37°C, 5% CO_2 overnight. Afterwards, colony counts were obtained. To obtain colonization density data from singly inoculated mice, nasal washes were quantified in the same manner as described above, and counts from the neomycin plates were used.

When attempting to quantify colonization density from mice inoculated with two competing strains, we noticed that the growth of the gentamicin-resistant strain on gentamicin-containing plates were inhibited by an unknown factor from the nasal wash. This occurred during both trials of the colonization assay and occurred in singly colonized mice who received the gentamicin resistant strain. This inhibition was not seen for the gentamicin-resistant strain growing on neomycin-containing plates from the singly inoculated mice. No inhibition was seen for either of the spectinomycin-resistant strains growing on either spectinomycin- or neomycin-containing plates. This inhibition was also not seen for any strain growing on any plate during plating of the inoculants. This phenomenon prevented us from obtaining accurate counts of the gentamicin-resistant strain from co-inoculated mice using the spot-plating method. However, since the inhibition was restricted to only gentamicin-containing plates, we developed an alternative method for quantifying the ratio of spectinomycin-resistant to gentamicin-resistant colonies isolated from co-inoculated mice using the samples plated on neomycin-containing plates. For each mouse, we picked 40-100 colonies from the spots on the neomycin-containing plates, grew them in THY + 5 μg/mL catalase at 37°C for 6-8 hours, then replicate-plated the cultures on TSA plates supplemented with 5 μg/mL catalase and containing either spectinomycin or gentamicin. After an overnight incubation at 37°C with 5% CO_2, each culture was scored for growth on spectinomycin or gentamicin. Cultures that showed growth on both antibiotics were excluded from further analysis. If none of the cultures from a mouse grew on a particular antibiotic, these data points were treated as being below the limit of detection and a count of 1 was used instead of 0. For each mouse, the output ratio was defined to be the count of cultures showing spectinomycin-resistant growth divided by the count of cultures showing gentamicin-resistant growth. We confirmed that neither of the spectinomycin-resistant strains could inhibit growth of the gentamicin-resistant strain or vice versa during growth on neomycin-containing plates or in co-culture in THY by repeating the above method on spot-plated samples from mixed cultures prepared in an identical fashion to those used to inoculate mice. Ratios obtained from the replicate-plating method agreed closely with ratios obtained from traditional counting of colonies on spectinomycin- and gentamicin-containing plates. The final competitive index calculation was performed using the following formula:

$$CI = R_{out} \frac{N_{gen,in}}{N_{spc,in}}$$

CI, competitive index; R_{out}, output ratio; $N_{gen,in}$, input CFU density of gentamicin-resistant strain; $N_{spc,in}$, input CFU density of spectinomycin-resistant strain.

References

1. Koressaar T, Remm M. 2007. Enhancements and modifications of primer design program Primer3. Bioinformatics 23:1289-91.
2. Untergasser A, Cutcutache I, Koressaar T, Ye J, Faircloth BC, Remm M, Rozen SG. 2012. Primer3--new capabilities and interfaces. Nucleic Acids Res 40:e115.

3. Sung CK, Li H, Claverys JP, Morrison DA. 2001. An *rpsL* cassette, janus, for gene replacement through negative selection in *Streptococcus pneumoniae*. Appl Environ Microbiol 67:5190-6.
4. Wang CY, Patel N, Wholey WY, Dawid S. 2018. ABC transporter content diversity in *Streptococcus pneumoniae* impacts competence regulation and bacteriocin production. Proc Natl Acad Sci U S A 115:E5776-E5785.
5. Liu X, Gallay C, Kjos M, Domenech A, Slager J, van Kessel SP, Knoops K, Sorg RA, Zhang JR, Veening JW. 2017. High-throughput CRISPRi phenotyping identifies new essential genes in *Streptococcus pneumoniae*. Mol Syst Biol 13:931.
6. Li Y, Thompson CM, Lipsitch M. 2014. A modified Janus cassette (Sweet Janus) to improve allelic replacement efficiency by high-stringency negative selection in Streptococcus pneumoniae. PLoS One 9:e100510.
7. Guiral S, Hénard V, Laaberki MH, Granadel C, Prudhomme M, Martin B, Claverys JP. 2006. Construction and evaluation of a chromosomal expression platform (CEP) for ectopic, maltose-driven gene expression in Streptococcus pneumoniae. Microbiology 152:343-9.
8. Chen YJ, Liu P, Nielsen AA, Brophy JA, Clancy K, Peterson T, Voigt CA. 2013. Characterization of 582 natural and synthetic terminators and quantification of their design constraints. Nat Methods 10:659-64.
9. Overkamp W, Beilharz K, Detert Oude Weme R, Solopova A, Karsens H, Kovács Á, Kok J, Kuipers OP, Veening JW. 2013. Benchmarking various green fluorescent protein variants in Bacillus subtilis, Streptococcus pneumoniae, and Lactococcus lactis for live cell imaging. Appl Environ Microbiol 79:6481-90.
10. Kochan TJ, Dawid S. 2013. The HtrA protease of Streptococcus pneumoniae controls density-dependent stimulation of the bacteriocin blp locus via disruption of pheromone secretion. J Bacteriol 195:1561-72.
11. Hiller NL, Janto B, Hogg JS, Boissy R, Yu S, Powell E, Keefe R, Ehrlich NE, Shen K, Hayes J, Barbadora K, Klimke W, Dernovoy D, Tatusova T, Parkhill J, Bentley SD, Post JC, Ehrlich GD, Hu FZ. 2007. Comparative genomic analyses of seventeen *Streptococcus pneumoniae* strains: insights into the pneumococcal supragenome. J Bacteriol 189:8186-95.
12. Jolley KA, Maiden MC. 2010. BIGSdb: Scalable analysis of bacterial genome variation at the population level. BMC Bioinformatics 11:595.
13. Huson DH, Bryant D. 2006. Application of phylogenetic networks in evolutionary studies. Mol Biol Evol 23:254-67.
